# Spatial heterogeneity of bacterial colonization across different gut segments following inter-species microbiota transplantation

**DOI:** 10.1186/s40168-020-00917-7

**Published:** 2020-11-18

**Authors:** Na Li, Bin Zuo, Shimeng Huang, Benhua Zeng, Dandan Han, Tiantian Li, Ting Liu, Zhenhua Wu, Hong Wei, Jiangchao Zhao, Junjun Wang

**Affiliations:** 1 State Key Laboratory of Animal Nutrition, College of Animal Science and Technology, China Agricultural University, Beijing, 100193 China; 2 Department of Laboratory Animal Science, College of Basic Medical Sciences, Third Military Medical University, Chongqing, 400038 China; 3 State Key Laboratory of Agricultural Microbiology, Key Laboratory of Agricultural Animal Genetics, Breeding, and Reproduction of the Ministry of Education, and Key Laboratory of Swine Genetics and Breeding of Ministry of Agriculture and Rural Affairs, College of Animal Sciences and Technology, Huazhong Agricultural University, Wuhan, Hubei 430070 China; 4 Department of Animal Science, Division of Agriculture, University of Arkansas, Fayetteville, AR 72701 USA

**Keywords:** Gut microbiota, Spatial heterogeneity, Different gut segments, Fecal microbiota transplantation, Whole-intestinal microbiota transplantation

## Abstract

**Background:**

The microbiota presents a compartmentalized distribution across different gut segments. Hence, the exogenous microbiota from a particular gut segment might only invade its homologous gut location during microbiota transplantation. Feces as the excreted residue contain most of the large-intestinal microbes but lack small-intestinal microbes. We speculated that whole-intestinal microbiota transplantation (WIMT), comprising jejunal, ileal, cecal, and colonic microbiota, would be more effective for reshaping the entire intestinal microbiota than conventional fecal microbiota transplantation fecal microbiota transplantation (FMT).

**Results:**

We modeled the compartmentalized colonization of the gut microbiota via transplanting the microbiota from jejunum, ileum, cecum, and colon, respectively, into the germ-free mice. Transplanting jejunal or ileal microbiota induced more exogenous microbes’ colonization in the small intestine (SI) of germ-free mice rather than the large intestine (LI), primarily containing Proteobacteria, Lactobacillaceae, and Cyanobacteria. Conversely, more saccharolytic anaerobes from exogenous cecal or colonic microbiota, such as Bacteroidetes, Prevotellaceae, Lachnospiraceae, and Ruminococcaceae, established in the LI of germ-free mice that received corresponding intestinal segmented microbiota transplantation. Consistent compartmentalized colonization patterns of microbial functions in the intestine of germ-free mice were also observed. Genes related to nucleotide metabolism, genetic information processing, and replication and repair were primarily enriched in small-intestinal communities, whereas genes associated with the metabolism of essential nutrients such as carbohydrates, amino acids, cofactors, and vitamins were mainly enriched in large-intestinal communities of germ-free mice. Subsequently, we compared the difference in reshaping the community structure of germ-free mice between FMT and WIMT. FMT mainly transferred LI-derived microorganisms and gene functions into the recipient intestine with sparse SI-derived microbes successfully transplanted. However, WIMT introduced more SI-derived microbes and associated microbial functions to the recipient intestine than FMT. Besides, WIMT also improved intestinal morphological development as well as reduced systematic inflammation responses of recipients compared with FMT.

**Conclusions:**

Segmented exogenous microbiota transplantation proved the spatial heterogeneity of bacterial colonization along the gastrointestinal tract, i.e., the microbiota from one specific location selectively colonizes its homologous gut region. Given the lack of exogenous small-intestinal microbes during FMT, WIMT may be a promising alternative for conventional FMT to reconstitute the microbiota across the entire intestinal tract.

Video Abstract

## Introduction

The mammalian gastrointestinal tract (GI-tract) harbors a highly complex and diverse microbial consortium that maintains a mutualistic relationship with the host, contributing to host development and health including the prevention of gut microbial dysbiosis [1]. Increasing evidence indicates that the targeted reconstitution of the gut microbiota is an ideal therapeutic strategy against gastrointestinal disorders [2–5]. Fecal microbiota transplantation (FMT) refers to the transfer of the fecal microbiota from a healthy donor into the gut of a diseased recipient, which restores the composition and functionality of the intestinal microbial community [6] and resists the colonization of pathogens [7]. For the past few years, FMT has been proposed to be a promising powerful therapy for diverse gastrointestinal disorders or immune-related pathologies, such as recurrent *Clostridium difficile* infection [8], inflammatory bowel disease [9], colitis [10], metabolic syndrome, and autism [11]. Several recent studies showed that FMT has the potential to trigger intestinal mucosal autophagy and alleviate gut barrier injury [12], improve growth performance [13], prevent early-weaning stress-induced diarrhea [14], as well as decrease the severity of porcine reproductive and respiratory syndrome [15].

The mammalian intestine is composed of a number of distinct microhabitats such as jejunum, ileum, cecum, and colon that selectively harbor characteristic microbes along the longitudinal axis of the intestinal lumens [16]. The small intestine (SI) including jejunum and ileum is a harsh microenvironment for microbial life because of the shorter transit time, lower pH values, and higher levels of oxygen and antimicrobials than the hindgut, and therefore, is dominated by rapidly growing facultative anaerobes such as Enterobacteriaceae and Lactobacteriaceae [17, 18]. In contrast, the large intestine (LI) including cecum and colon dominantly hosts a number of saccharolytic anaerobes such as Bacteroidaceae, Prevotellaceae, Rikenellaceae, Lachnospiraceae, and Ruminococcaceae [17, 18]. The small-intestinal microbiota is mainly responsible for simple carbohydrates and amino acid metabolism, while the large-intestinal community is more favorable for the fermentation of complex polysaccharides [17–19].

In most studies about microbiota transplantation, human feces have been the primary materials for transplantation because they are relatively easy and non-invasive to collect without many ethical issues [20, 21]. Of note, fecal materials, the excreted residue of the digestive tract, are different from the whole intestinal digesta. Previous studies demonstrated that the fecal community contains the vast majority of microbial species and functionality derived from the large-intestinal community rather than the small-intestinal community [19, 22]. A single fecal sample fails to capture the overall variation in bacterial colonization along the whole GI-tract. Therefore, FMT might only reconstitute the LI microbiome, without much effect on the dysbiosis of the SI microbiome.

Given the spatial heterogeneity of bacterial distribution across distinct intestinal sections due to physiological variations including nutrient concentrations [23], chemical gradients [24], intestinal architecture, as well as host immunity [25], we hypothesized that transplantation of exogenous microbiota from a specific gut segment of the donor leads to niche-specific colonization of its corresponding niche (gut location) of the recipient. We further hypothesized that the conventional FMT only directly modulates the large-intestinal microbiota of recipients while transplanting the combined microbiota from both SI and LI can be more effective in correcting dysbiosis in other GI-tract locations (e.g., SI).

In this study, to test these hypotheses, we first characterized the niche-specific colonization of the gut microbiota by transplanting the luminal microbiota obtained from distinct gut segments of pigs, including jejunum, ileum, cecum, and colon, into germ-free mice. We subsequently compared the differences in reshaping the gut microbiota structure of the germ-free mice between the whole-intestinal microbiota transplantation (WIMT) and the conventional FMT from the pigs. In addition, we also evaluated the effects of different microbiota transplantation on the intestinal development and immune responses of recipients. The germ-free mice as the most developed model system for understanding the interaction between the host and its microbiota were selected as recipients in this study. Most investigations on germ-free mice extensively selected exogenous fecal microbiota from human beings as donors to generate the “humanized mice” [26]. However, the “humanized mice” might be missing the effects of the SI microbiota in these studies. The pig as a human-sized and omnivorous animal is a more promising animal model over other non-primate models for studying the microbiota transplantation since richer intestinal contents are more readily captured across the entire GI-tract in pigs. Besides, pigs have the highest similarity with human beings in terms of physiology, anatomy, behavioral patterns, and gut microbiota [27–29]. Therefore, we finally chose pigs as donors in the present study. The findings of this study will provide insights into the mechanism by which exogenous microbiota transplantation reconstitutes the intestinal microbiota of recipients and advocate the use of WIMT as a promising alternative for the conventional FMT in restoring mammalian gut microbial balance in other GI-tract locations.

## Materials and methods

### Preparation of microbiota suspension of donors

Duroc × Landrace × large white crossbred barrows, with a mean body weight of 50 kg, provided by FengNing Swine Research Unit of China Agricultural University (Academician Workstation in Chengdejiuyun Agricultural and Livestock Co., Ltd), were used as donors for the microbiota transplantation of different gut segments. According to the criteria for donor selection described by Hu et al. [30], pigs used in this study had no diarrhea or other digestive infections and had not been administered with any antibiotics or other drugs for at least 2 months prior to digesta collection. We simultaneously collected the digesta from different gut segments of the donor pigs, including jejunum, ileum, cecum, and colon, as well as fresh feces from the rectum. The microbiota suspension was prepared as described below. Briefly, fresh content from the same gut segment was pooled from different pigs, homogenized, diluted 5-fold in sterile potassium phosphate buffer (0.1 M, pH 7.2) containing 15% glycerol (v/v), then immediately dispensed to cryotubes, and stored at –80°C. The whole-intestinal microbiota suspension was generated by mixing the digesta from jejunum, ileum, cecum, and colon according to the ratio of their volumes (jejunum:ileum:cecum:colon = 2:3:1:4). The remaining contents and tissues from different gut sections were snap-frozen in liquid nitrogen and stored at −80°C until subsequent analysis.

### Niche-specific microbiota transplantation from distinct gut segments

**Fig. 1:**
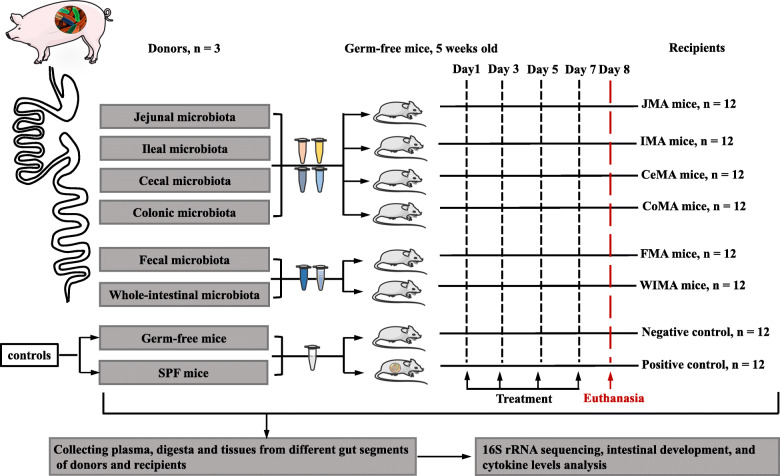
The timeline of treatments and sample collection. JMA mice: jejunal microbiota-associated mice; IMA: ileal microbiota-associated mice; CeMA: cecal microbiota-associated mice; CoMA: colonic microbiota-associated mice; FMA: fecal microbiota-associated mice; WIMA: whole-intestinal microbiota-associated mice; SPF: specific-pathogen-free mice

Five-week-old germ-free mice and specific-pathogen-free (SPF) Kunming mice with similar body weights were bred at the Department of Laboratory Animal Science in the Third Military Medical University in Chongqing, China. At first, a total of 48 germ-free mice were selected as recipients in this study to characterize the niche-specific colonization across different gut segments. These germ-free mice were randomly allocated to four groups with 12 mice per group. These mice were inoculated orally with 0.3 mL of jejunal, ileal, cecal, or colonic microbial suspension, respectively, once every other day for 7 days. Additional 2 mL aliquots were spread on the fur of each mouse. In addition, 12 germ-free mice and 12 SPF mice gavaged with sterile saline were used as controls. As a result, a total of six treatments were included in the first experiment: jejunal microbiota-associated (JMA) mice, ileal microbiota-associated (IMA) mice, cecal microbiota-associated (CeMA) mice, colonic microbiota-associated (CoMA) mice, germ-free mice, and SPF mice. Subsequently, we performed a follow-up experiment to investigate the difference in reshaping the gut microbiota structure between WIMT and the conventional FMT. A total of 24 germ-free mice were randomly assigned to 2 groups with 12 mice per group to generate fecal microbiota-associated (FMA) mice and whole-intestinal microbiota-associated (WIMA) mice inoculated with the fecal and whole-intestinal suspension, respectively. All the mice were euthanized at 6 weeks of age by intracardiac injection of sodium pentobarbital, and the digesta and tissues of jejunum, ileum, cecum, and colon, as well as feces, were collected and stored at −80°C. The experimental design and sample collection are illustrated in Fig. 1.

### DNA extraction and 16S rRNA sequencing

Total DNA was extracted from the intestinal contents of donors and recipients using the QIAamp® Fast DNA Stool Mini Kits (Qiagen Ltd., Germany) according to the manufacturer’s instructions. The V3-V4 region of the 16S rRNA gene was amplified with universal primers 338F (ACTCCTACGGGAGGCAGCAG) and 806R (GGACTACHVGGGTWTCTAAT). The cycling conditions of PCR reactions were 95 °C for 3 min; 29 cycles of 95 °C for 30 s, 55 °C for 30 s, and 72 °C for 45 s; and a final extension at 72 °C for 10 min. After purification and quantification, the PCR products were pooled into equal molar amounts and sequenced on an Illumina MiSeq sequencer to generate paired-end reads of 300 bp.

### Microbiota data analysis

Raw sequences were analyzed using the latest version of the QIIME2 platform (version 2.4) as previously described by Wang et al. [13]. Initial reads were quality filtered, denoised, assembled, and chimeric sequences were removed using Deblur [31], which generates unique amplicon sequence variants (ASVs) or bacterial features instead of clustering similar sequences into traditional operational taxonomic units [31]. Subsequently, we used the Greengenes reference database classifier (version 13-8) for the classification of bacterial features with a threshold of 100% sequence similarity. Alpha and beta diversities were also calculated in QIIME2. To examine the factors (e.g., the donor and the recipient gut segment) shaping the colonization of the pig-derived gut microbiota in the recipient gut, we performed permutational multivariate analysis of variance (PERMANOVA, with 1000 Monte Carlo permutations) based on Bray-Curtis and Jaccard distances with the Adonis function available in the package “vegan” in R software [32]. Differentially abundant features between groups were identified using linear discriminant analysis (LDA) effect size (LEfSe) analysis [33]. Only taxa with average relative abundances greater than 0.01% were included in LEfSe. The package “VennDiagram” of R software was used to assess the proportion of porcine-associated microbes that were successfully transplanted into different gut niches of recipient mice in this study. Bar plots and heat maps were visualized using the “ggplot2” and “pheatmap” packages of R software (version 3.3.1) (https://www.r-project.org/), respectively. The predicted metagenomes and function of the gut microbiota were inferred by using PICRUSt2 (https://github.com/picrust/picrust2). Differentially abundant KEGG pathways between groups were calculated using STAMP (version 2.1.3).

### Total bacterial population determination by qPCR assay

Total DNA was extracted from the intestinal digesta samples as mentioned above. PCR amplification was carried out with the total bacterial primers 338F (ACTCCTACGGGAGGCAGCAG) and 518R (ATTACCGCGGCTGCTGG). The qPCR was conducted with the Roche LightCycler® 96 Real-time PCR system (Roche, Sweden). The reaction mixture (25 μL) contained 1.5 μL forward and 1.5 μL reverse primers, 12.5 μL 2 × TB GreenTM Premix Ex TaqTM II (Takara, Japan), 1 μL template DNA, and 8.5 μL ddH_2_O. The reaction protocol consisted of one initial denaturation at 95 °C for 10 min, 40 cycles of denaturation at 95 °C for 10 s, 60 s at the appropriate annealing temperature (60 °C), and extension at 72 °C for 10 s. The copy numbers of the total bacteria were calculated using the corresponding standard curve. The standard curve was generated as described by Han et al. [34]. Briefly, the target standard plasmid of total bacteria was constructed, and a series of 10-fold dilution (10^9^ to 10^1^ copies/μL) of the plasmids DNA for total bacteria were used to generate its respective standard curve with the logarithm of target copy numbers as the abscissa and the Ct values as the ordinate. The gene copy numbers were calculated using the equation as follows: (DNA concentration (μg/μL) × 6.0233 × 10^23^ copies/mol)/(DNA size (bp) × 660 × 10^6^). All PCR reactions were performed in duplicate.

### Intestinal morphology, goblet cells, and cell apoptosis

After fixation with 4% paraformaldehyde for 24 h, the jejunal and ileal samples were embedded in paraffin, sectioned, and stained with hematoxylin and eosin for histological analysis. Then, the Alcian Blue and Periodic Acid Schiff staining assay were performed to measure the number of intestinal acidic and neutral mucins secreted by goblet cells, respectively. Determination of villus height, crypt depth, and the number of goblet cells and glycoproteins, were performed using CaseViewer software (version 2.2) at × 200 magnification. The extent of cell apoptosis in the jejunum and ileum of recipients was evaluated using a commercial TUNEL staining kit (Roche, Sweden) according to the manufacturer’s instructions, and subsequently judged via a fluorescence microscope. For each sample, at least 10 villi or crypts or sections were counted.

### Plasma inflammatory profiles

The concentrations of pro-inflammatory cytokines (IFN-γ, IL-12p70, IL-1β, IL-5, IL-6, KC/GRO, and TNF-α) and anti-inflammatory cytokines (IL-2, IL-4, and IL-10) were determined using mouse V-PLEX kits (Meso Scale Discovery, USA) according to the manufacturer’s directions.

### Statistical analysis

Data were analyzed using SPSS 22.0 for Windows (SPSS Inc., Chicago, USA). All parametric data were analyzed using unpaired Student’s *t* test or one-way ANOVA with Tukey’s post hoc test. All non-parametric data were analyzed using the Mann-Whitney *U* test or Kruskal-Wallis test. *P* values for multiple comparisons were adjusted with a false discovery rate (FDR) according to Benjamini and Hochberg [35]. The corrected *P* values below 0.05 were considered statistically different. Data were expressed as means and standard error of the mean (SEM).

## Results

### Spatial heterogeneity for exogenous bacterial colonization across different gut segments

A total of 4,896,764 high-quality reads were generated with an average of 16,655 reads in each sample and were assigned into 2729 bacterial features based on 100% sequence similarity. These features were then classified into 19 phyla, 45 classes, 103 orders, 118 families, 202 genera, and 118 species. We first examined if the microbiota transplantation follows a niche-specific pattern by inoculating germ-free mice with digesta collected from different gut segments of pigs.

#### Niche-specific colonization across different gut segments

**Fig. 2:**
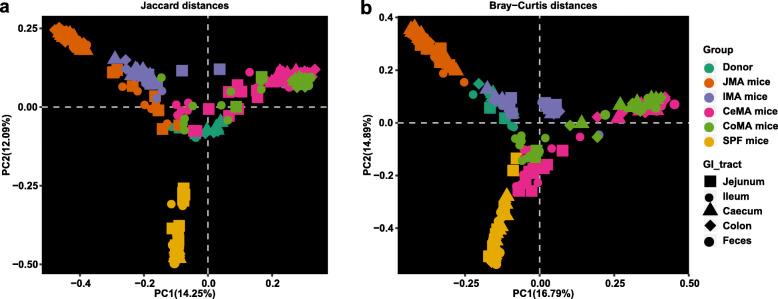
Gut microbiota structure of recipient mice, SPF mice, and donor pigs. Principal coordinate analysis (PCoA) plots based on the Jaccard distance (**a**) and Bray-Curtis distance (**b**) showed distinct clusters in donor and mouse samples. JMA mice: jejunal microbiota-associated mice; IMA: ileal microbiota-associated mice; CeMA: cecal microbiota-associated mice; CoMA: colonic microbiota-associated mice; SPF: specific-pathogen-free mice

At the community level, we calculated beta (e.g., Jaccard and Bray-Curtis distance) and alpha (e.g., the number of observed bacterial features and Shannon index) diversities of the gut microbiota in different locations along the GI-tract in germ-free mice receiving microbiota transplantation from the jejunum (JMA), ileum (IMA), cecum (CeMA), and colon (CoMA) of pigs. Principal coordinates analysis (PCoA) plots showed that microbial community structures were dramatically different among different groups of recipient mice (Fig. 2a, b). PERMANOVA based upon Bray-Curtis and Jaccard distance indicated that the gut segment of both the donor and the recipient significantly affected the reconstruction of the exogenous gut microbiota in the recipient gut, with about 40% variation attributed to the donor (*F* = 33.44, *P* = 0.001). The gut segment of the recipient also explained about 6% of the variation in reshaping the gut microbiota of recipients (*F* = 3.38, *P* = 0.001).

**Fig. 3:**
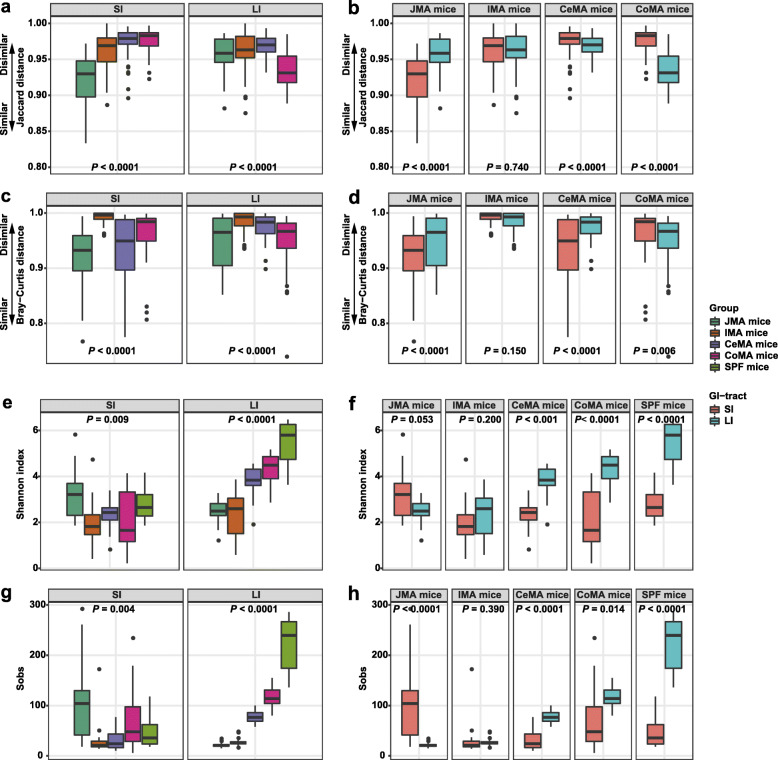
Differences in beta and alpha diversities of gut microbiota in recipient mice and SPF mice. Differences in the Jaccard distance from the recipient to the donor among different groups (**a**) and between SI and LI of recipients (**b**). Differences in the Bray-Curtis distance from the recipient to the donor among different groups (**c**) and between SI and LI of recipients (**d**). Differences in the community diversity (Shannon index) among different groups (**e**) and between SI and LI of recipients (**f**). Differences in the community richness (sobs) among different groups (**g**) and between SI and LI of recipients (**h**). Jejunal and ileal samples of recipients were pooled into small-intestinal samples. Cecal, colonic, and fecal samples of recipients were pooled into large-intestinal samples. JMA mice: jejunal microbiota-associated mice; IMA: ileal microbiota-associated mice; CeMA: cecal microbiota-associated mice; CoMA: colonic microbiota-associated mice; SPF mice: specific-pathogen-free mice; SI: small intestine; LI: large intestine

There was also a significant difference in the community structure between the recipient SI and LI within each treatment except for IMA mice (Additional file 1: Fig. S1a–e, Fig. S2a–e). The community composition bar plots also showed that the microbiota composition in the recipient jejunum and ileum (SI) was similar, while that among the recipient cecum, colon, and feces (LI) was similar (Additional file 1: Fig. S3a–c). Therefore, for subsequent analysis, jejunal and ileal samples of recipients were pooled into small-intestinal samples while cecal, colonic, and fecal samples of recipients were pooled into large-intestinal samples. Significant differences in the distance from the recipient to the donor among different recipients were observed (Fig. 3a, c, *P* < 0.0001). In the small-intestinal samples, the Jaccard and Bray-Curtis distances from the recipient to the donor jejunum were smallest in JMA mice. In the large-intestinal samples, CoMA mice had the smallest Jaccard and Bray-Curtis distances to the donor’s colon. The small-intestinal samples of JMA mice had significantly smaller Jaccard and Bray-Curtis distances to jejunal samples of their donors than large-intestinal samples (Fig. 3b, d, *P* < 0.0001). On the contrary, the large-intestinal samples of CeMA mice had significantly smaller Jaccard distances to their donors than small-intestinal samples (Fig. 3b, *P* < 0.001). The large-intestinal samples of CoMA had remarkably smaller Jaccard and Bray-Curtis distances from their donors than small-intestinal samples (Fig. 3b, d, *P* < 0.001). This suggests that the corresponding gut region of the recipient receiving the microbiota transplantation from a particular gut segment could have a more similar microbiota structure to its donor gut segment.

We subsequently determined the differences in the 16S gene copy number of total bacteria in all samples using quantitative PCR. Significant differences in the total bacterial population were observed in different intestinal segments of the donor, among which the counts in the jejunum was the smallest and the number in the colon was the greatest (Additional file 1: Fig. S4a, *P* < 0.05). Compared with other groups, JMA mice had the smallest total bacterial population both in the SI and LI (Additional file 1: Fig. S4b, *P* < 0.0001). With respect to alpha diversities of overall microbiota communities, significant differences among different recipients were also observed (Fig. 3e, g, *P* < 0.001). In the small-intestinal samples, JMA mice had the highest number of observed features and the Shannon index. In the large-intestinal samples, these indexes of CeMA and CoMA mice were significantly higher than JMA and IMA mice. Consistent with the SPF mice, the Shannon index (*P* < 0.001) and the number of observed features (*P* < 0.05) were dramatically higher in the LI of both CeMA and CoMA mice than those in the SI (Fig. 3f, h). The absolute counts of total bacteria in the LI of all groups were also significantly greater than that in the SI of these mice (Additional file 1: Fig. S4c, *P* < 0.0001). No significant changes in alpha diversity were observed between the SI and LI of IMA mice (Fig. 3f, h). However, greater microbial diversity including the Shannon index (*P* = 0.053) and the number of observed features (Sobs) (*P* < 0.0001) was detected in the SI compared to that in the LI of JMA mice in this study (Fig. 3f, h). These findings showed that transplanting the microbiota from a particular gut segment could selectively increase the microbial diversity of its corresponding gut region of recipients.

#### Niche-associated bacterial taxa

Transplanting the exogenous microbiota from different gut segments also dramatically affected the microbial composition of recipient mice (Additional file 1: Fig. S3). The reconstituted community composition in recipient mice also significantly differed from that in donors and SPF mice (Additional file 1: Fig. S3). We next estimated the extent of donor microbiota colonization using the Venn diagram analysis, which revealed shared features between donors and recipients. In this study, these shared bacteria were considered as microbes that were successfully transplanted into the recipient intestine (Additional file 1: Fig. S5-S8; Additional file 2: Table S1a-S1d).

In JMA mice (Additional file 1: Fig. S5; Additional file 2: Table S1a), 60 jejunum-associated features were only transplanted into the SI and six jejunum-associated features had greater relative abundances in the SI than those in the LI, with dominant families being Lactobacillaceae (*n* = 8), Lachnospiraceae (*n* = 6), and Ruminococcaceae (*n* = 6). Seventeen features were associated with Proteobacteria (*n* = 17) and four features within Cyanobacteria (*n* = 4). In IMA mice (Additional file 1: Fig. S6; Additional file 2: S1b), 16 ileum-associated features were colonized in the SI, with one feature associated with Cyanobacteria, four features within Lactobacillaceae, and three features within Lachnospiraceae. These data showed that the exogenous small-intestinal microbiota might be more inclined to colonize in the recipient SI relative to the recipient LI.

On the contrary, 19 cecum-associated features only colonized in the LI of CeMA mice and 17 features were enriched in the LI compared to those in the SI (Additional file 1: Fig. S7; Additional file 2: S1c). These microbes were associated with Ruminococcaceae (*n* = 9), Erysipelotrichaceae (*n* = 6), Coriobacteriaceae (*n* = 5), Prevotellaceae (*n* = 4), and Veillonellaceae (*n* = 3). Consistently, a total of 76 features that only appeared in the LI and 23 features enriched in the LI of CoMA mice could be attributed to the donor community (Additional file 1: Fig. S8; Additional file 2: S1d). These microbes were classified as Lachnospiraceae (*n* = 32), Ruminococcaceae (*n* = 26), Erysipelotrichaceae (*n* = 7), Mogibacteriaceae (*n* = 6), and Prevotellaceae (*n* = 6). Therefore, in contrast to the small-intestinal microbiota, the large-intestinal community members of donors might prefer to colonize in the LI rather than the SI of the recipients.

#### Niche-specific microbial metabolic pathways across different gut segments

We subsequently inferred the functions of microbiota using PICRUST2 to infer the functional differences between the exogenous microorganisms residing in the recipient SI and LI. We obtained a total of 6909 KEGG orthologs, which were classified into 41 categories of gene pathways at level 2 against the KEGG database. The results of STAMP also demonstrated similar niche-specific colonization patterns in the predicted microbial gene pathways of the recipient intestine. The metabolic potential of the SI microbiota significantly differed from that of LI microbiota of the recipients (Additional file 1: Fig. S9, *P* < 0.05). The SI microbiota of JMA mice were enriched with genes in 10 pathways compared with the LI microbiota (Additional file 1: Fig. S9a, *P* < 0.05). These pathways included a poorly characterized pathway, nucleotide metabolism, genetic information processing, transcription, replication and repair, metabolism of cofactors and vitamins, metabolic diseases, metabolism of terpenoids, and polyketides, translation, and immune system diseases. In the IMA mice, the proportions of genes involved in six pathways (nucleotide metabolism, carbohydrate metabolism, genetic information processing, transcription, replication and repair, and metabolism of terpenoids and polyketides) were dramatically increased in the SI community members (Additional file 1: Fig. S9b, *P* < 0.05). In agreement with the above-mentioned results, the microbial metabolic pathways in the SI of donors were more likely to be transferred into the recipient SI rather than the LI.

Compared with the small-intestinal community of recipients, genes related with 12 functional pathways were significantly increased in the large-intestinal community of CeMA mice, including a poorly characterized pathway, carbohydrate metabolism, transcription, energy metabolism, cellular processes and signaling, enzyme families, metabolism of cofactors and vitamins, metabolism of other amino acids, environmental adaptation, metabolism of terpenoids and polyketides, immune system diseases, as well as glycan biosynthesis and metabolism (Additional file 1: Fig. S9c, *P* < 0.05). In addition, greater relative abundances of genes related with nine pathways were also observed in the large-intestinal community of CoMA mice than those in the small-intestinal community, which comprised carbohydrate metabolism, transcription, energy metabolism, cellular processes, and signaling, metabolism of cofactors and vitamins, environmental adaptation, metabolism of terpenoids and polyketides, and folding sorting and degradation (Additional file 1: Fig. S9d, *P* < 0.05). These data suggested that the microbial gene pathways in the LI of donors had a greater tendency to be transferred into the recipient LI instead of the SI, which was also similar to the aforementioned results about the community composition.

### Differences in reconstituting the gut microbiota structure between FMT and WIMT

Based on the above-mentioned results, the bacterial community derived from a certain gut segment might prefer to reside in its corresponding gut regions in the recipients. Feces as the excreted residue contain the majority of microbial species and functionality in the LI [19]. We hypothesized that only part of the donors’ large-intestinal microorganisms could be transferred into the recipient LI by FMT, leaving the small-intestinal microbiota unaffected. Therefore, we next conducted a follow-up test to examine whether transplanting the whole-intestinal microbiota was more efficient at reshaping the gut microbiota structure compared with the conventional FMT.

#### Overall gut microbiota composition of FMA and WIMA mice

**Fig. 4:**
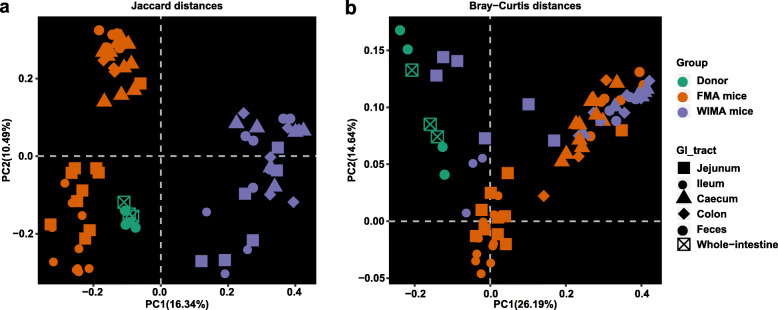
Gut microbiota structure of FMA mice, WIMA mice, and donor pigs. Principal coordinate analysis (PCoA) plots based on the Jaccard distance (**a**) and Bray-Curtis distance (**b**) showed distinct clusters in donor and mouse samples. FMA mice: fecal microbiota-associated mice; WIMA mice: whole-intestinal microbiota-associated mice

PCoA plots and Bar plots indicated that the community structure in WIMA mice was significantly different from that in FMA mice (Fig. 4a, b; Additional file 1: Fig. S3a-c), which was confirmed again by PERMANOVA with an *F* value of 13.55 explaining about 17% of the variation (*P* = 0.001). Distinct segregations of the community structure were also found between the SI and LI of FMA and WIMA mice (PERMANOVA *F* = 5.74, *R*^2^ = 0.27, *P* = 0.001; Additional file 1: Fig. S10a–d).

**Fig. 5:**
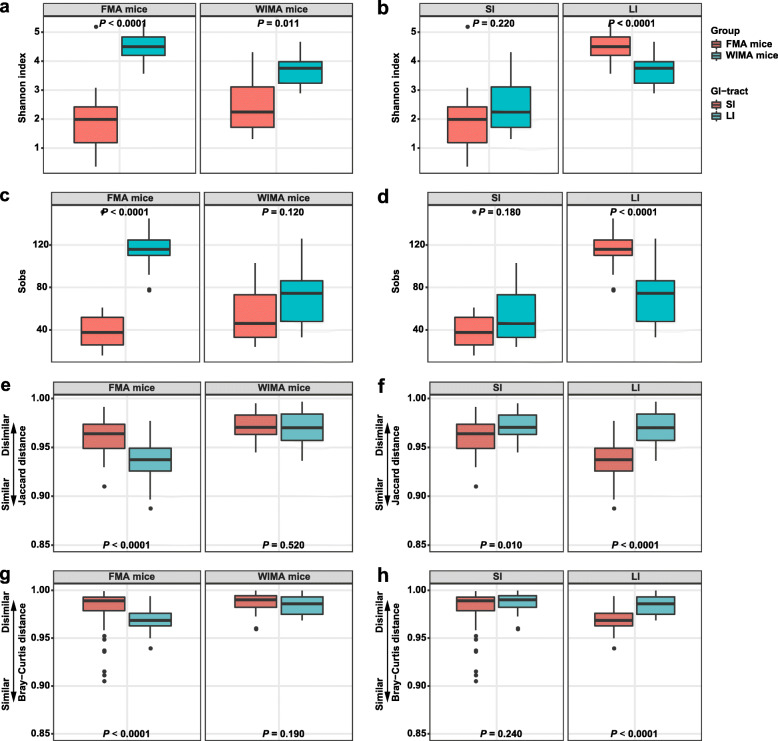
Differences in alpha and beta diversities of gut microbiota in FMA and WIMA mice. Differences in the community diversity (Shannon index) between SI and LI of recipients (**a**) and between FMA mice and WIMA mice (**b**). Differences in the community richness (sobs) between SI and LI of recipients (**c**) and between FMA mice and WIMA mice (**d**). Differences in the Jaccard distance from the recipient to the donor between SI and LI of recipients (**e**) and between FMA mice and WIMA mice (**f**). Differences in the Bray-Curtis distance from the recipient to the donor between SI and LI of recipients (**g**) and between FMA mice and WIMA mice (**h**). Jejunal and ileal samples of mice were pooled into small-intestinal samples. Cecal, colonic, and fecal samples of mice were pooled into large-intestinal samples. FMA mice: fecal microbiota-associated mice; WIMA mice: whole-intestinal microbiota-associated mice; SI: small intestine; LI: large intestine

A greater large-intestinal microbial diversity (*P* < 0.0001, Fig. 5a, c) and a smaller distance between large-intestinal samples and donors (*P* < 0.0001, Fig. 5e, g) were observed in FMA mice, again suggesting a more similar community structure between the recipient LI and donor feces compared with the recipient SI. The counts of total bacteria in the LI were also significantly greater than that in the SI of FMA or WIMA mice (Additional file 1: Fig. S11b, *P* < 0.0001). Compared with WIMA mice, FMA mice had a greater total bacteria load in the SI and LI (Additional file 1: Fig. S11c, *P* < 0.0001). On the other hand, the LI of FMA mice had higher numbers of observed features and Shannon indices as well as smaller Bray-Curtis and Jaccard distances from the recipient LI to donors than the LI of WIMA mice (*P* < 0.0001, Fig. 5b, d, f, h). Interestingly, WIMA mice had a larger Jaccard distance from the recipient SI to donors with no significant change in the Bray-Curtis distance compared with FMA mice (*P* < 0.01, Fig. 5f, h).

#### Differentially abundant exogenous microbes successfully transplanted into FMA and WIMA mice

**Fig. 6:**
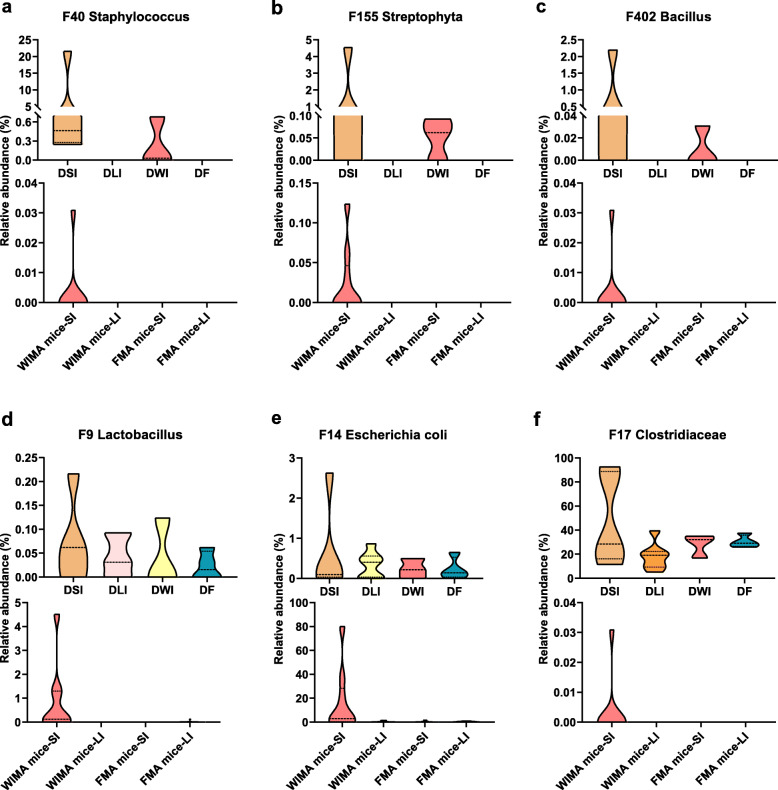
Small intestinal-specific microbes in donors that successfully colonized the SI of WIMA mice. Bacterial features that were either absent (**a**, **b**, **c**) or less abundant in the donor feces than in the donor whole intestine (**d**, **e**, **f**) only colonizing the SI of WIMA mice, were referred to as “small intestine-specific microbes” during WIMT. Jejunal and ileal samples of mice were pooled into small-intestinal samples. Cecal, colonic, and fecal samples of mice were pooled into large-intestinal samples. DSI: donor small-intestine; DLI: donor large-intestine; DWI: donor whole-intestine; DF: donor feces; SI: small intestine; LI: large intestine; FMA mice: fecal microbiota-associated mice; WIMA mice: whole-intestinal microbiota-associated mice

In FMA mice, 50 fecal-derived features were colonized only in the LI of FMA mice and 12 fecal-derived features were more abundant in the LI than those in the SI, most of which belonged only to the cecal and/or colonic communities (Additional file 1: Fig. S12; Additional file 2: Table S1e). These microbes were primarily classified into Ruminococcaceae, Prevotellaceae, and Lachnospiraceae, suggesting that FMT primarily grafted a portion of large-intestinal microorganisms of donor pigs into the LI of recipient mice whereas only a few small-intestinal microbes were successfully transferred. As to the WIMA mice, more pig-derived features were still transplanted into the LI rather than the SI (39 vs. 13, Additional file 1: Fig. S13; Additional file 2: Table S1f). However, a greater proportion of porcine microbes were transplanted into the SI of WIMA mice than those of FMA mice (Fig. 6; Additional file 1: Fig. S13; Additional file 2: Table S1f, 17 vs. 7). We next examined the niche-specific bacterial features that demonstrated colonization preference in certain niches. Figure 6 shows several bacterial features that were more abundant in the donor SI than in the donor LI. Consistently, these features were either absent (e.g., F40 (*Staphylococcus*, Fig 6a), F155 (Streptophyta, Fig. 6b), and F402 (*Bacillus*, Fig. 6c)), or less abundant in the donor feces than in the donor whole intestine (e.g., F9 (*Lactobacillus*, Fig. 6d), F14 (*Escherichia coli*, Fig. 6e), and F17 (Clostridiaceae, Fig. 6f)). After transplantation, these features only colonized the SI of WIMA mice (Fig. 6) and thus were referred to as “small intestine-specific microbes” during WIMT. They were not observed in the SI of FMA mice, even when present in the donor feces, suggesting the niche preference of these features.

**Fig. 7:**
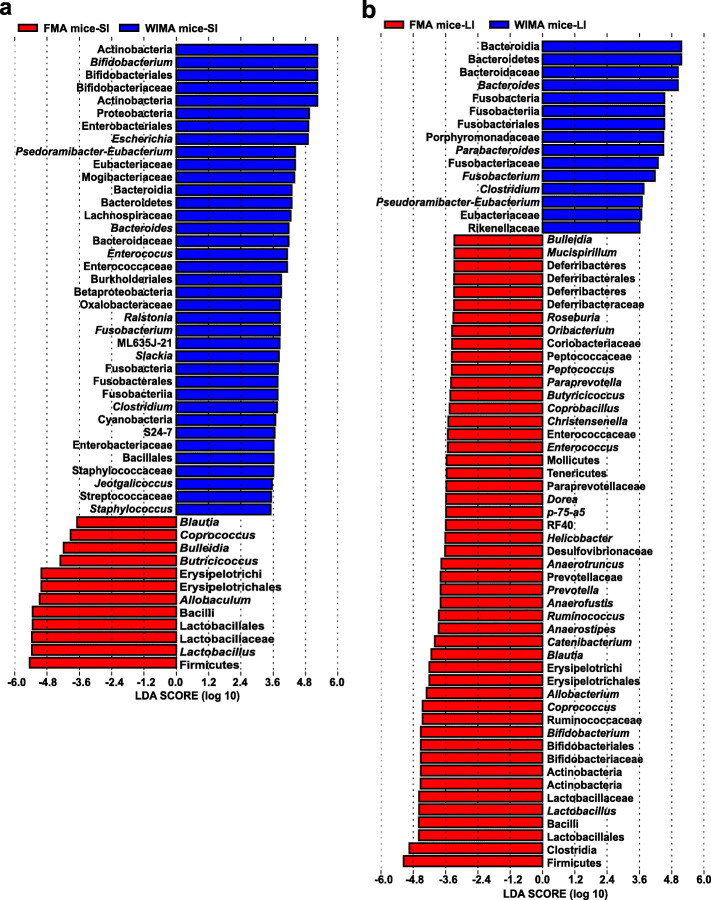
Differentially abundant taxa between FMA and WIMA mice. Histograms of a linear discriminant analysis (LDA) score (threshold ≥ 2) in small-intestinal samples (**a**) and large-intestinal samples (**b**) are plotted. Jejunal and ileal samples of recipients were pooled into small-intestinal samples. Cecal, colonic, and fecal samples of recipients were pooled into large-intestinal samples. FMA mice: fecal microbiota-associated mice; WIMA mice: whole-intestinal microbiota-associated mice; SI: small intestine; LI: large intestine

Next, LEfSe analysis identified differentially abundant bacterial taxa in the small- and large-intestinal microbiota between FMA and WIMA mice (Fig. 7). In the recipient SI, a total of 37 taxa were significantly enriched in WIMA mice while only 12 taxa were enriched in FMA mice (Fig. 7a). The relative abundances of Actinobacteria, Proteobacteria, Cyanobacteria, Bacteroidetes, and Fusobacteria were significantly higher in the SI of WIMA mice while the phylum Firmicutes were more abundant in the SI of FMA mice. WIMA mice also had higher proportions of the families Bifidobacteriaceae, Eubacteriaceae, Lachnospiraceae, Bacteroidaceae, Enterococcaceae, Enterobacteriaceae, Staphylococcaceae, and Streptococcaceae but lower proportions of Lactobacillaceae compared to FMA mice. At the genus level, *Bifidobacterium*, *Escherichia*, *Bacteroides*, *Enterococcus*, *Fusobacterium*, *Clostridium*, and *Staphylococcus* were enriched in the SI of WIMA mice whereas *Blautia*, *Coprococcus*, *Butyricoccus*, and *Lactobacillus* were more abundant in the SI of FMA mice. However, in the recipient LI, more bacterial taxa were significantly enriched in FMA mice compared with WIMA mice (49 vs. 15, Fig. 7b). Relative abundances of the phyla Bacteroidetes and Fusobacteria, the family Eubacteriaceae, as well as the genera *Bacteroides*, *Fusobacterium*, and *Clostridium*, were also obviously greater in the LI of WIMA mice than those of FMA mice. Compared with the WMT, FMT significantly increased relative abundances of the phyla Firmicutes, Actinobacteria, and Deferribacteres in the LI. The families Lactobacillaceae, Bifidobacteriacea, Ruminococcaceae, Prevotellaceae, Desulfovibrionaceae, Paraprevotellaceae, Enterococcaceae, Peptococcaceae, Coriobacteriaceae, and Deferribacteraceae were also more abundant in the LI of FMA mice than those of WIMA mice. In addition, FMA mice presented markedly greater proportions of the genera *Lactobacillus*, *Bifidobacterium*, *Coprococcus*, *Allobacterium*, *Blautia*, *Prevotella*, *Helicobacter*, *Enterococcus*, *Christensenella*, *Butyricoccus*, *Paraprevotella*, *Peptococcus*, and *Roseburia* in the LI than WIMA mice. These findings again suggested that WIMT might increase the colonization of small-intestinal microbes than the conventional FMT.

#### Differentially abundant microbial metabolic pathways between FMA and WIMA mice

We next performed a functional analysis of microbiota using PICRUST2 to identify differentially represented microbial metabolic pathways between the microbiota residing in the gut of FMA and WIMA mice (Additional file 1: Fig. S14). The microbial community in the SI of WIMA mice had significantly higher relative abundances of genes associated with 11 pathways compared with FMA mice (Additional file 1: Fig. S14a, *P* < 0.05), including translation, carbohydrate metabolism, metabolism of terpenoids and polyketides, environmental adaptation, transport and catabolism, membrane transport, cell communication, metabolism of cofactors and vitamins, immune system, glycan biosynthesis and metabolism, and transcription. As to the large-intestinal microbiota, significantly greater relative abundances of genes involved in 11 pathways (a poorly characterized pathway, carbohydrate metabolism, transcription, energy metabolism, metabolism of cofactors and vitamins, amino acid metabolism, metabolism of other amino acids, infectious diseases, metabolism of terpenoids and polyketides, lipid metabolism, and immune system diseases) were observed in WIMA mice, while genes associated with seven pathways (membrane transport, biosynthesis and biodegradation of secondary metabolites, cellular processes and signaling, replication and repair, environmental adaptation, xenobiotics biodegradation and metabolism, and immune system) were remarkably enriched in FMA mice (Additional file 1: Fig. S14b, *P* < 0.05). A greater number of microbial functional pathways were enriched in SI and LI of recipients generated by WIMT compared with FMT.

### Small-intestinal epithelium physiology

To characterize the effects of different microbiota transplantation on the small-intestinal development of recipients, we measured the villus height, crypt depth, and the number of mucins and glycoproteins, as well as the extent of cell apoptosis in the recipient jejunum and ileum. No significant changes in villus height, crypt depth, cell apoptosis, the number of goblet cells or glycoproteins, were observed in the SI of any recipient mice from the niche-specific transplantation (i.e., JMA, IMA, CeMA, and CoMA mice) (Additional file 1: Fig. S15, *P* > 0.05).

**Fig. 8:**
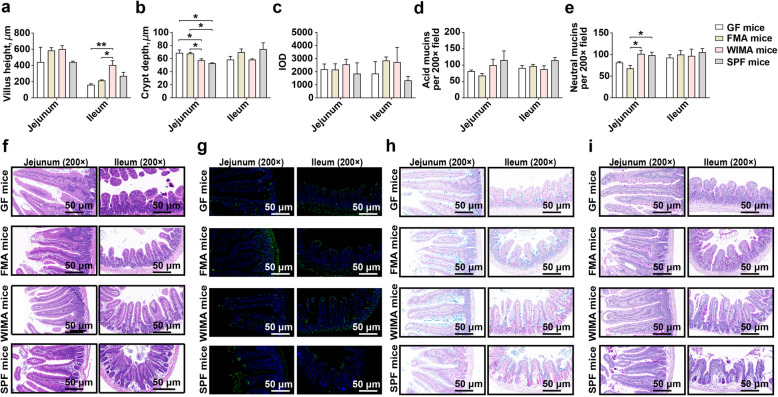
The development of small-intestinal epithelial morphology of germ-free, FMA, WIMA, and SPF mice. Differences in the villus height (**a**), crypt depth (**b**), the number of apoptotic positive cells (**c**), and the number of acid mucins (**d**) and neutral mucins (**e**) in the jejunum and ileum among different groups are presented. The hematoxylin and eosin staining of the jejunum and ileum of different groups (**f**). The TUNEL staining of the jejunum and ileum of different groups (**g**), the green fluorescent cell nuclei were selected as the apoptotic positive cells. The Alcian Blue staining of the jejunum and ileum of different groups (**h**), the acidic mucins were stained in blue. The Periodic Acid-Schiff staining of the jejunum and ileum of different groups (**i**), the neutral mucins were stained in magenta red. Data are shown as mean±SEM. *P < 0.05, **P < 0.01. GF mice: germ-free mice, FMA mice: fecal microbiota-associated mice; WIMA mice: whole-intestinal microbiota-associated mice; SPF mice: specific-pathogen-free mice; IOD: integrated optical density

However, a significant increase in the ileal villus height and a significant decrease in the jejunal crypt depth were observed in WIMA mice than those in germ-free and FMA mice (Fig. 8a, b, f, *P* < 0.05). Besides, the jejunal crypt depth was significantly deeper in germ-free and FMA mice than SPF mice (Fig. 8b, f, *P* < 0.05). TUNEL staining showed no obvious difference in the number of apoptotic positive cells in small-intestinal epithelium between FMA and WIMA mice (Fig. 8c, g, *P* > 0.05). The counts of neutral mucins secreted by the goblet cells were significantly increased in the jejunum of WIMA mice than that of FMA mice (Fig. 8e, i, *P* < 0.05). FMA mice had less neutral mucins in the jejunum than SPF mice (Fig. 8e, i, *P* < 0.05) with no difference between WIMA and SPF mice (Fig. 8e, i, *P* > 0.05).

### Plasma inflammatory profiles

We next sought to characterize the differences in plasma inflammatory indices among different groups of mice (Additional file 1:Fig. S16). No obvious influence on the levels of IFN-γ, IL-12p70, TNF-α, and IL-4 was observed (Additional file 1: Fig. S16a, e.g., i, *P* > 0.05). However, the concentration of IL-1β was significantly lower in germ-free, JMA, IMA, CeMA, and CoMA mice compared with SPF mice (Additional file 1: Fig. S16b, *P* < 0.05). A higher concentration of IL-1β was seen in the plasma of CeMA mice than that of germ-free mice (Additional file 1: Fig. S16b, *P* < 0.01). Transplanting the microbiota from different gut segments induced a lower concentration of IL-5 in the plasma of recipients compared with germ-free mice (Additional file 1: Fig. S16c, *P* < 0.05). The plasma concentration of IL-5 in CeMA, CoMA, and SPF mice were significantly lower than that in JMA and IMA mice (Additional file 1: Fig. S16c, *P* < 0.001). The highest concentrations of IL-6 and IL-2 were observed in the plasma of CeMA mice compared to other groups (Additional file 1: Fig. S16d, h, *P* < 0.05). The concentration of KC/GRO was higher in the plasma of CeMA mice than that of SPF mice (Additional file 1: Fig. S16f, *P* < 0.05). The plasma of CeMA mice and CoMA mice had a significant increment in the level of IL-10 compared to JMA and SPF mice (Additional file 1: Fig. S16j, *P* < 0.05).

**Fig. 9:**
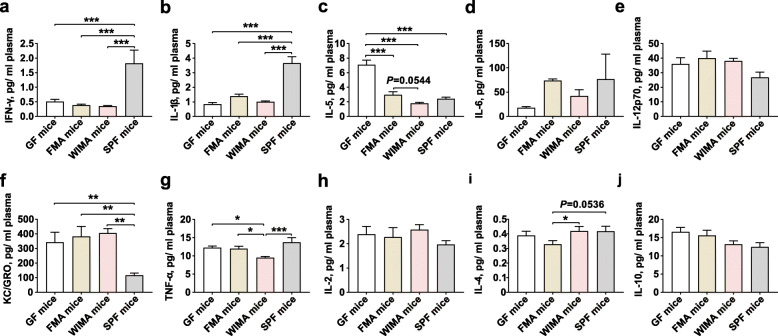
Plasma inflammatory profiles of germ-free, FMA, WIMA, and SPF mice. Differences in concentrations of IFN-γ (**a**), IL-1β (**b**), IL-5 (**c**), IL-6 (**d**), IL-12p70 (**e**), KC/GRO (**f**), TNF-α (**g**), IL-2 (**h**), IL-4 (**i**), and IL-10 (**j**) among different groups are presented. Data are shown as mean±SEM. *P < 0.05, **P < 0.01, ***P < 0.001. GF mice: germ-free mice, FMA mice: fecal microbiota-associated mice; WIMA mice: whole-intestinal microbiota-associated mice; SPF mice: specific-pathogen-free mice

We next assessed the differences in secretion levels of inflammatory cytokines in the plasma of WIMA and FMA mice. The decreased concentrations of IFN-γ and IL-1β but the higher level of KC/GRO were observed in the plasma of germ-free, FMA, and WIMA mice compared to SPF mice (Fig. 9a, b, f, *P* < 0.01). The concentrations of IL-5 and TNF-α were lower in the plasma of WIMA mice compared with germ-free and FMA mice (Fig. 9c, g, *P* < 0.10). In contrast, the concentration of IL-4, an anti-inflammatory cytokine, was significantly greater in the plasma of WIMA mice than that of FMA mice (Fig. 9i, *P* < 0.05).

## Discussion

Over the past decade, the effectiveness of FMT in the therapy of a series of gut infections has attracted much attention to its potential application [9]. Nonetheless, the mammalian intestine contains diverse microbial niches with compartmentalized physiological variations, such as jejunum, ileum, cecum, colon, feces, etc., which are responsible for the segmented distribution of the intestinal microorganisms [16–18]. Although it is now acknowledged that the bacterial communities are significantly discrete among different microhabitats, it remains unclear whether the community membership derived from a particular gut niche only selectively identifies and resides in its homologous gut location. The fecal community contains a large proportion of microbial species of the large-intestinal microbiota with sparse small-intestinal microbes [19, 22]. Therefore, we speculated that transplanting the microbiota derived from both SI and LI might be more effective for reshaping the entire intestinal microbiota, particularly the SI microbiota, and the treatment of gut diseases than the conventional FMT. Here, for the first time, our study demonstrated the spatial heterogeneity of exogenous bacterial colonization through inter-species microbiota transplantation from pig to germ-free mice. Our results showed that microorganisms and microbial functional genes derived from one particular intestinal segment were more inclined to colonize its homologous gut niche of the recipient. While FMT administration, which has been used as a surrogate of the LI, transferred a part of LI-derived microorganisms into the recipient LI, it transferred very few SI-derived microbes to the recipient SI. In contrast, compared with FMT, WIMT, which also contains contents from the SI, could contribute more to the colonization of small-intestinal microbes as well as further facilitate intestinal development and health.

### Niche-specific colonization of exogenous microbiota across different gut segments

In our study, we first observed that overall community structure differed among different groups of recipients receiving segmented microbiota suspension consistent with a previous study colonizing germ-free mice with microbial communities from diverse gut environments [36].

Transferring the small-intestinal microbiota led to similar community structures between SI and LI of recipients. This could be attributed to an insufficient bacterial diversity and richness of the exogenous small-intestinal community compared to the exogenous large-intestinal community. Compared with other recipients, JMA mice had the greatest small-intestinal microbial diversity and a more similar small-intestinal microbial structure with their donor, while the greatest large-intestinal microbial diversity and a more similar large-intestinal microbial structure with their donor were observed in CoMA mice. Interestingly, we also observed a greater community diversity and a more similar community structure between recipients and donors in the recipient SI originating from the exogenous jejunal microbiota transplantation relative to the recipient LI. Unfortunately, we did not observe an increasing trend in the gut microbiota diversity in the SI of recipients colonized by the exogenous ileal microbiota in our trial likely due to an intrinsically lower diversity of the input ileal community compared to other exogenous sources. On the contrary, as for these recipients receiving the exogenous cecal and colonic microbiota transplantation, the community diversity in the recipient LI and the similarity between the recipient LI and the donor was significantly greater than those in the recipient SI. Physiological differences along the longitudinal axis of the intestinal tract consist of oxygen availability, acidic gradient, and nutrient substrates, as well as host immune activities, all of which comprehensively drive the spatial heterogeneity of the gut bacterial community [17, 18] and might explain the niche-specific development patterns of overall community structures and diversities in the recipient gut receiving exogenous microbiota derived from different gut segments. But beyond that, the total bacteria load in the LI within JMA or IMA mice were significantly greater than that in the SI, which is somewhat inconsistent with the results of alpha microbial diversity. This could be explained by the difference in analytical tools for quantification. The total bacterial load measured by qPCR only referred to the absolute total counts of bacteria in the overall microbial community, irrespective of bacterial species. The alpha microbial diversity was estimated by the number of observed features representing the community richness as well as the Shannon index representing the community richness and evenness. The former was calculated by the number of observed feature species but the latter by the number and the relative abundance of different species [37]. This also indicated that JMA and IMA mice had the higher small-intestinal community richness and evenness than their large-intestinal microbiota despite the opposite outcomes of the total bacterial load.

Despite that the recipient mice did not harbor a microbial community equal to the donor pig, our results presented the niche-specific colonization of exogenous bacterial species across different gut segments in recipients. For small-intestinal microbiota-associated recipients, a greater number of exogenous bacterial taxa were transplanted into the recipient SI whereas few microbes colonized in the recipient LI. These exogenous microbes enriched in the recipient SI consisted of a higher relative abundance of the phylum Proteobacteria and the family Lactobacillaceae relative to the recipient LI. The mammalian foregut is more acidic, has a faster transit time and higher gradients of oxygen and antimicrobials compared with the LI [17]. As a consequence, the small-intestinal community membership mainly contains fast-growing facultative anaerobes such as Proteobacteria and Lactobacillaceae, which are equipped to resist the synergistic effect of bile acids and antimicrobial peptides [16]. Members of the phylum Cyanobacteria, containing a consortium of aerotolerant bacteria which are considered to be the ancestors of algae and chloroplasts and obtain energy through photosynthesis [38], were also more inclined to colonize in the recipient SI but were rarely found in the recipient LI. Several previous studies have also reported that Cyanobacteria are prevalent in the small intestine of piglets [39, 40] and were related to the occurrence of necrotizing enterocolitis [39]. Besides, members within Proteobacteria as commensals in the gut include an array of opportunistic pathogens, such as Enterobacteriaceae (e.g., *Enterobacter* species), which harbor the significant pathogenic potential to induce the intestinal inflammation [41–43]. It also means that the commensal pathogens originating from the exogenous microbiota might be more likely to invade the recipient SI.

Conversely, a greater proportion of exogenous microbes were successfully colonized in the LI of large-intestinal microbiota-associated recipients than those in the recipient SI. It is well acknowledged that lower concentrations of antimicrobials, slower transit time, and less available simple carbohydrates result in greater bacterial density in the hindgut [36, 44]. The community membership in the hindgut is primarily responsible for the fermentation of complex non-digestible polysaccharides, therefore, facilitating the dominance of fermentative polysaccharide-degrading anaerobes, notably Bacteroidaceae and Ruminococcaceae [17]. In our study, we observed that a portion of pig-derived microbes capable of degrading indigestible carbohydrates leading to the production of short-chain fatty acids (SCFAs), including Bacteroidetes [45], Prevotellaceae [46], and some families of Firmicutes such as Veillonellaceae [47], Lachnospiraceae [48], and Ruminococcaceae [49], had a greater preference to invade the recipient LI relative to the recipient SI. Families Erysipelotrichaceae and Mogibacteriaceae under the phylum Firmicutes, which are designated as “obesity” bacterial taxa positively correlated with the production of secondary bile acids [50–52], also exhibited greater relative densities in the recipient LI receiving the introduction of exogenous large-intestinal communities than those in the recipient SI. Microbes within Erysipelotrichaceae were also positively correlated with a number of parameters involved in carbohydrate digestion including dietary carbohydrate and fiber content and SCFAs production while negatively correlated with fat digestibility and protein metabolism [53–55]. Previous studies also have reported these microbes are significantly increased in the hindgut of pigs [46, 56, 57]. Altogether, these observations indicate that saccharolytic anaerobic bacteria from exogenous large-intestinal communities are primarily enriched in the recipient LI instead of the recipient SI, which might result in increased microbial capabilities to produce SCFAs to maintain host health.

Nonetheless, we noted the obvious individual differences among the replicates of recipients in our study. The pig-derived microbes successfully colonizing the intestine of recipients were only detected in a minority of replicates from JMA and IMA mice, particularly IMA mice, while more individuals from CeMA and CoMA mice were successfully transplanted with pig-derived microbes but were still very varied. The input microbiota in the SI, with lower bacterial population and diversity than that in the LI, might be less competitive and co-adaptable to their recipients along with an inter-species transplantation, thus leading to a higher intra-individual variability among their recipients compared with these recipients receiving exogenous large-intestinal microbes.

The host and its gut microbiota are linked by microbial gene functions derived from the microorganisms. In this study, our results demonstrated the similar spatial heterogeneity of the predicted microbial gene pathways of the recipient intestine in agreement with the aforementioned observations associated with the community composition. We observed that microbial gene functions derived from exogenous small-intestinal communities were more likely to be transferred into the recipient SI, whereas gene functions from exogenous large-intestinal communities had a greater tendency to be transferred into the recipient LI. Particularly, our data revealed that the abundances of genes related to nucleotide metabolism, genetic information processing, replication and repair, xenobiotics biodegradation and metabolism, and immune system were significantly enriched in small-intestinal bacterial communities of recipients as compared to large-intestinal communities. The enrichment of these functional pathways may reflect increased genetic information transmission and expression as well as augmented mediation of immune system diseases in the recipient SI. However, the functional alterations of the large-intestinal community in recipients were characterized by significantly increased abundances of functions associated with amino acid metabolism, lipid metabolism, carbohydrate metabolism, energy metabolism, metabolism of cofactors and vitamins, metabolism of other amino acids, transcription, and cellular processes and signaling. Zhao and colleagues also have reported that microorganisms in the LI exhibit higher proportions of functions related to metabolic pathways of important nutrients than those in the SI such as carbohydrates and energy metabolism [19]. It has been demonstrated that intestinal microorganisms are of great importance for amino acid catabolism and dietary energy uptake responsible for the production of diverse bacterial metabolites such as ammonia and SCFAs [58, 59]. Gill et al. [60] also found the distal gut microbiota was enriched for a variety of COGs associated with biosynthesis of essential amino acids and vitamins. Furthermore, Zhang and his colleagues reported that these functional pathways were positively correlated with microbes of Bacteroidetes, Lachnospiraceae, and Ruminococcaceae [46], which were enriched microbes in the recipient LI in our study.

On the other hand, the immune system plays a fundamental role in sustaining the symbiotic relationship of the host with these highly diverse commensal microbes [61]. Our study indicated that transplanting different exogenous communities significantly affected the systemic inflammatory profiles of recipient mice, as reflected by relatively higher concentrations of two pro-inflammatory cytokines (IL-1β and IL-6) in the plasma of CeMA recipients than those of germ-free mice. At the same time, the increased concentration of IL-2 and IL-10 was observed in the plasma of recipients originating from the introduction of the exogenous cecal and colonic microbiota compared to other groups of recipients. Intestinal microorganisms promote the release of IL-1β and IL-6 by macrophages and dendritic cells in intestinal lymphoid tissues and the periphery [62–64]. Mice lacking IL-6 receptor or IL-1 receptor 1 induce a lower frequency of IL-10-producing B cells and reduce the IL-10 secretion compared to wild-type mice [65]. IL-2 is acutely required to maintain Treg cells and immunological homeostasis in the GI-tract and the low doses of IL-2 has been used as a potential therapy for inflammatory diseases [66, 67]. IL-2 can be induced selectively by the IL-1β production [66] and has been also validated as a positive regulator in the IL-10 production in activated intestinal innate lymphoid cells [68]. The release of IL-10 plays a pivotal role in the differentiation of regulatory B cells responsible for the suppression of excessive inflammation [69]. Burrello and colleagues [10] have reported that exogenous FMT exerts multiple effects on restraining intestinal inflammation and initiating the restoration of intestinal homeostasis through simultaneously triggering several anti-inflammatory pathways associated with IL-10 production by innate and adaptive immune cell subsets. Here, increased release of IL-1β, IL-6, as well as IL-2 and IL-10 in the plasma from recipient mice receiving exogenous large-intestinal communities reflected a similar effect in our study. Notably, the introduction of the exogenous cecal microbiota resulted in the highest levels of IL-1β, IL-6, and IL-2 in the plasma of cecal-associated recipient mice, which might be because of the greatest bacterial richness in the input cecal community. In line with our results, Rosser and colleagues also found that antibiotic-treated mice with a low microbial density exhibited reduced secretion of IL-10, IL-1β, and IL-6 by splenocytes compared with control mice [65]. Moreover, transplanting the exogenous cecal microbiota induced a greater level of KC/GRO in the plasma of CeMA mice compared with SPF mice in the present study. The murine chemokine CXCL1 (KC/GRO) released via tissue macrophages or mast cells is one of the major chemoattractants responsible for neutrophil recruitment which serves as a critical early step in regulating tissue inflammation [70]. IL-5 is considered a proinflammatory cytokine closely associated with allergic disorders through regulating the differentiation and the release of eosinophils [71]. In our study, the production of IL-5 was significantly decreased in the plasma of recipients receiving the administration of cecal and colonic microbiota transplantation relative to other groups of recipients. These outcomes implicate a potential beneficial effect of the exogenous large-intestinal microbiota transplantation on host anti-inflammation.

### WIMT contributes more to the colonization of the exogenous gut microbiota across the entire intestinal tract relative to the conventional FMT

The fecal microbial community contains the majority of microbial species and functionality derived from the large-intestinal community with rare small-intestinal microbes [19], supporting observations in our study. On the basis of the niche-specific colonization pattern of exogenous communities, as we described above, the large-intestinal microbes of donors might prefer to colonize in the recipient LI rather than the recipient SI. Moreover, we observed here that the large-intestinal community of FMA mice was more similar to donor feces compared with the small-intestinal community, which are consistent with the results in recipients receiving the large-intestinal microbiota transplantation in our study. Therefore, we speculate that the administration of FMT might not be the ideal approach to reshaping the bacterial community structure of germ-free recipients because of the absence of small-intestinal microbes from donors, compared with transplanting the whole-intestinal microbiota including jejunal, ileal, cecal, and colonic microbiota.

In this study, at the overall community level, FMA mice, rather than WIMA mice, had more similar small-intestinal and large-intestinal microbiota to their donor microbiota. We believe this result is due to the differences in the complexities of the donor microbiota. FMT used in this study only contained the majority of LI-derived microbes of the donor with sparse SI-derived microbes, “simpler” than WIMT, which was composed of microbiota from different locations of the SI and LI of the donor. In this case, the SI of WIMA mice was more likely to selectively accept small-intestinal microbes from whole-intestinal transplants while donor large-intestinal microbes were alternatively colonized in the LI of WIMA mice. As a result, the difference in the microbiota structure between WIMA mice and the donor whole-intestinal materials was greater than that between FMA mice and the donor fecal materials. This finding might also reflect a potential defect of FMT since the small-intestinal community structure of recipients should not be assumed similar to the donor feces but similar to the donor SI.

At the microbial community composition, FMT primarily transferred a part of exogenous large-intestinal microbes, such as Ruminococcaceae, Paraprevotellaceae/Prevotellaceae, as well as *Blautia* and *Roseburia* within Lachnospiraceae, into the recipient LI whereas fewer small-intestinal microbes were successfully transferred. These bacteria taxa were also enriched in the recipient LI receiving exogenous large-intestinal communities in our study, which are capable of degrading refractory carbohydrates to produce SCFAs. Fecal materials, the excreted residue of the digestive tract, fail to capture the overall variation in bacterial colonization along the entire GI-tract because of the absence of the SI microbiota. In many previous studies using humanized mice, microbes successfully colonizing the recipient intestine also primarily originated from the large-intestinal microbiome of donors, such as Lachnospiraceae, Prevotellaceae, and Ruminococcaceae, with few small-intestinal microbes colonized [72–75]. These outcomes are in line with our study and indicate the missing effects of the small-intestinal microbiota of donors in these FMT studies.

However, performing WIMT resulted in a greater number of exogenous small-intestinal microbes colonizing the recipient SI compared to FMT, including Actinobacteria, Cyanobacteria, Proteobacteria, Fusobacteria, which were more abundant in small-intestinal communities of donors. Members of families Enterobacteriaceae and Enterococcaceae were also more prevalent in the SI of WIMA mice. We observed that some small intestine-specific microbes derived from the donor whole intestine only colonized the recipient SI instead of the recipient LI during WIMT, such as F40, F155, F402, F9, F14, and F17, suggesting the niche preference of these features. These features were primarily classified as Lactobacillaceae, Cyanobacteria, Enterobacteriaceae, Clostridiaceae, Staphylococcaceae, and Bacillaceae. These outcomes were similar to the small-intestinal microbiota composition of mice receiving exogenous small-intestinal communities. In brief, these findings demonstrated that WIMT contributes more to the colonization of exogenous small-intestinal microorganisms in the recipient SI than the conventional FMT.

Furthermore, our results suggested that compared with FMT, WIMT was more favorable to the development of intestinal microbial gene functions of recipients. We observed that a greater number of microbial functional pathways were enriched in SI and LI of recipients by the administration of WIMT compared with FMT. In particular, genes associated with several microbial metabolic pathways of indispensable nutrients including carbohydrate metabolism, glycan biosynthesis and metabolism, metabolism of cofactors and vitamins, energy metabolism, amino acid metabolism, and lipid metabolism were significantly up-represented in the intestine of recipient mice receiving WIMT than those of mice receiving FMT. The enrichment of these microbial pathways might reflect an enhancement of the microbiota-mediated catabolic and biosynthetic capacity of essential nutrients in the recipient intestine receiving WIMT relative to conventional FMT. Of note, all the differential gene functions identified in this study were inferred from the bacterial composition by PICRUST, which is a quick approach to gaining insights into metabolic pathways. However, more experiments are needed to verify these pathways by qPCR and/or RNAseq to determine if these genes are really expressed and abundant.

The intestinal epithelium, equipped with the largest mucosal surface of the body, is adjacent to intestinal resident microorganisms, and permanent interactions with each other play important parts in enhancing barrier function to prevent pathogen invasion [76]. The intestinal villus capillaries of germ-free mice developed more poorly from weaning to adulthood compared to conventional mice, revealing that the intestinal bacterial colonization is essential for villus development of [76]. A previous study also suggested that exogenous FMT could contribute to the development of intestinal villus morphology [77]. Surprisingly, we observed that performing WIMT further increased the ileal villus height and decreased the small-intestinal crypt depth compared with the conventional FMT. A protective mucus layer, comprising diverse gel-forming mucin glycoproteins secreted by goblet cells, overlays the intestinal epithelium and provides the frontline host defense against pathogen attachment [78, 79]. Intestinal commensal bacteria can directly regulate functions of goblet cells and mucus layers depending on the delivery of host-derived bioactive factors produced by epithelial or lamina propria cells [79]. Hu and colleagues [77] found that the exogenous fecal microbiota suspension results in a significant upregulation of mRNA and protein expressions of Mucin2 in recipient piglets. Mucins are classified into neutral and acidic subtypes and acidic mucin depending on the types of polysaccharide chains. Neutral mucins appear to occur in greater quantities in gastric mucosa and small-intestinal epithelium, whereas acidic mucins are throughout the whole intestinal tract and predominate in the large intestine [75, 78]. In this study, compared with the conventional FMT, WIMT further increased the amount of neutral mucins in the jejunum of recipients. These observations indicate that the administration of WIMT may exert greater beneficial effects on the development of intestinal epithelium structure and barrier function as compared to the conventional FMT.

In addition, we observed the decreased concentrations of IFN-γ and IL-1β in the plasma of germ-free, FMA, and WIMA mice compared to SPF mice. However, the murine chemokine CXCL1 (KC/GRO) responsible for neutrophil recruitment was increased in the plasma of germ-free, FMA, and WIMA mice compared to SPF mice [70]. Mice receiving WIMT exhibited lower plasma concentrations of IL-5 and TNF-α but higher levels of IL-4 compared with mice receiving FMT. The marked reduction of pro-inflammatory cytokines in the serum appears to be emerging as an important target for the treatment of intestinal inflammation, such as TNF-α, IL-1β, and IFN-γ [80]. IL-1β and TNF-α are also considered prime pro-inflammatory cytokines secreted by macrophages, which can cause cell death and promote full activation of macrophages [81]. IL-5 is another pro-inflammatory cytokine leading to allergic symptoms via the generation of eosinophils [71]. IL-4 acts as one multifunctional anti-inflammatory cytokine that plays central roles in the regulation of T cell proliferation, gene expression, and in preventing cell apoptosis [82]. Hence, alterations of the secretion of these cytokines in this study might demonstrate that exogenous WIMT could reduce systematic inflammatory responses in the host better than FMT.

It should be noted, however, that another previous study indicated that the small intestine mucus layer of germ-free mice colonized with mouse cecal microbiota required about 5 weeks of microbial colonization to become normally detached [83]. Thus, 1-week colonization conducted in this study might be a relatively shorter time span for systemic inflammatory response and small-intestinal mucus layer formation of recipients and might not be able to fully reflect the profound effects on host homeostasis. How the microbiota from segmented intestinal tracts affect immunological and mucus systems of recipients warrants further exploration through a longer-period microbiota conventionalization of germ-free animals.

In the present study, it is almost impossible to collect intestinal materials from different GI-tract locations of humans to test our hypothesis. Pigs have been considered as the “best” biomedical model for studying human diseases because their microbiotas are more human-like than mice [27–29]. In addition, the most abundant intestinal contents are more readily captured across the entire GI-tract in pigs. Therefore, we chose to use pigs as a model to test our hypothesis as a proof-of-concept. However, one limitation of this study was the lack of extensive colonization of the donor’s microbiota in the recipients. Cross-species microbiota transplantation could be challenging likely because of the short adaptation period of the donor microbiota in the recipients. Nevertheless, previous studies using non-native microbiota transplantation showed that about half of the fecal microbiota in human microbiota-associated mice could be successfully attributable to the human donor source [72, 73]. Besides, the dominant microbiota phyla and genera existing in pig donors could also be successfully conserved in the pig microbiota-associated mice following inter-species microbiota transplantation [84–86]. In our study, despite the small number of donor bacterial colonization in the recipients, the data still supported our hypothesis, i.e., the microbiota from one specific gut location selectively colonizes its homologous gut region. Moreover, the clear differences in the microbial structure between the SI and LI of donor pigs could still be reproduced in their recipient mice. These findings are in line with previous studies where cross-species (e.g., human to mice, pigs to mice) microbiota transplantation resulted in limited microbiota colonization in the recipients, but significant phenotypes were still effectively reproduced [84–89]. Owing to the limited number of germ-free facilities, we were not able to include a positive control group with the conventional mouse donor transplantation. Future studies are desired to include such a group, which would likely result in better colonization in the recipients and better support our hypothesis.

## Conclusions

**Fig. 10:**
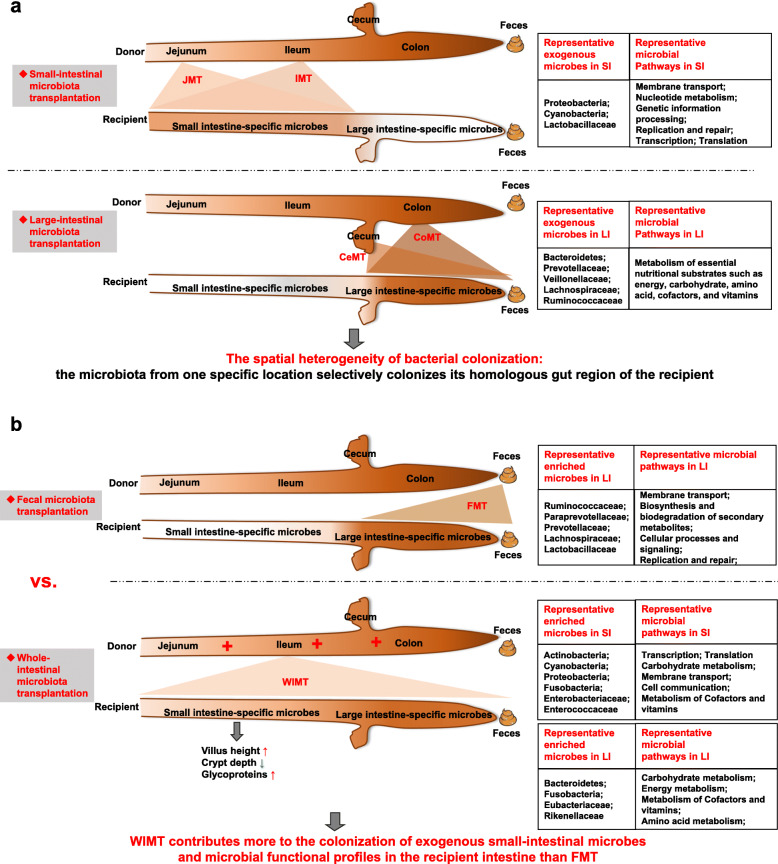
Integrative diagram showing the main results obtained from the present work. JMT: jejunal microbiota transplantation; IMT: ileal microbiota transplantation; CeMT: cecal microbiota transplantation; CoMT: colonic microbiota transplantation; FMT: fecal microbiota transplantation; WIMT: whole-intestinal microbiota transplantation; SI: small intestine; LI: large intestine

Taken together, segmented exogenous microbiota transplantation induced the spatial heterogeneity of bacterial colonization along the GI-tract that the microbiota derived from one particular gut segment selectively colonizes its homologous gut region of the recipient. The introduction of exogenous jejunal or ileal microbiota resulted in a greater number of exogenous microbes invading the recipient SI instead of the recipient LI, primarily containing members of Proteobacteria, Lactobacillaceae, and Cyanobacteria. On the contrary, more saccharolytic anaerobes derived from exogenous large-intestinal communities capable of degrading indigestible carbohydrates, such as Bacteroidetes, Prevotellaceae, Veillonellaceae, Lachnospiraceae, and Ruminococcaceae, had a greater preference to reconstitute in the LI rather than the SI of recipients. Similar segmented colonization patterns of exogenous microbial gene functions in the recipient intestine were also observed. Genes related to nucleotide metabolism, genetic information processing, replication and repair, and immune system were primarily enriched in small-intestinal communities, whereas genes associated with the metabolism of essential nutritional substrates such as energy, carbohydrate, amino acid, cofactors, and vitamins were mainly enriched in large-intestinal communities of recipients. We also demonstrated that FMT principally transferred a part of LI-derived microorganisms into the recipient gut with only a few SI-derived microbes. Compared with the conventional FMT, WIMT might contribute more to the colonization of exogenous small-intestinal microbes and microbial functional profiles in the recipient intestine as well as be more beneficial to intestinal development and host health. Our study contributes to a better understanding of the reconstitution of exogenous microorganisms by FMT and provides novel insights for the use of WIMT as a promising alternative therapy for conventional FMT in mammals (summarized in Fig. 10). Nevertheless, only a part of donor microbes successfully colonized in the intestine of germ-free mice in the present study due to an inter-species transplantation from pigs to mice. With this in view, we propose that the follow-up study in the future should be conducted using mice and humans as additional donors alongside pigs for diminishing the effects of non-native microbiota transplantation. Other germ-free animal hosts that are more human-like beings could be better developed and used, such as gnotobiotic pigs. Just as important, experiments on assessing the changes in immunological and mucus systems of recipients remain to be further addressed through a longer-period conventionalization of germ-free animals with microbiota from segmented intestinal tracts. As for the practical application of the WIMT, non-invasive approaches are being developed such as a customized multichannel catheter [90] and swallowable bio-sampling capsules programmed to sample luminal contents [91]. Moreover, an in vitro dynamic continuous culture system, which allows for strict and stable control of bacterial growth conditions to make it similar to those of the human intestine [92], would be a very powerful approach to produce standardized cultivated cocktails that include bacterial isolates from SI and LI of donors.

## Supplementary information

**Additional file 1: Supplementary figures. Figure S1.** Gut microbiota structure of recipient mice, SPF mice, and donor pigs. Principal coordinate analysis (PCoA) plots based on Bray-Curtis distances in jejunal microbiota-associated mice, T1 (a), ileal microbiota-associated mice, T2 (b), cecal microbiota-associated mice, T3 (c), colonic microbiota-associated mice, T4 (d), and specific-pathogen-free mice (e). D: Donor; J: Jejunum; I: Ileum; Ce: Cecum; Co: Colon; F: Feces. **Figure S2.** Gut microbiota structure of recipient mice, SPF mice, and donor pigs. Principal coordinate analysis (PCoA) plots based on Jaccard distances in jejunal microbiota-associated mice, T1 (a), ileal microbiota-associated mice, T2 (b), cecal microbiota-associated mice, T3 (c), colonic microbiota-associated mice, T4 (d), and specific-pathogen-free mice (e). D: Donor; J: Jejunum; I: Ileum; Ce: Cecum; Co: Colon; F: Feces. **Figure S3.** Gut microbiota composition among different groups of donors and mice. Abundant phyla (a), families (b), and genera (c) in the gut microbiota of different groups of donors and mice. Only genera with average relative abundance greater than 1% were shown. Data are shown as means in each group, D: Donor; J: Jejunum; I: Ileum; Ce: Cecum; Co: Colon; F: Feces; WI: Whole intestine; T1: Jejunal microbiota-associated mice; T2: Ileal microbiota-associated mice; T3: Cecal microbiota-associated mice; T4: Colonic microbiota-associated mice; T5: Fecal microbiota-associated mice; T6: Whole-intestinal microbiota-associated mice; SPF: Specific-pathogen-free mice. **Figure S4.** Total bacterial population in recipient mice, and SPF mice, that were determined by quantitative PCR. Differences in the copy numbers of the total bacteria (log10 copies/g wet digesta) among donors (a), among different groups (b) and between SI and LI of recipients (c). Jejunal and ileal samples of recipients were pooled into small-intestinal samples. Cecal, colonic, and fecal samples of recipients were pooled into large-intestinal samples. JMA mice: Jejunal microbiota-associated mice; IMA: Ileal microbiota-associated mice; CeMA: Cecal microbiota-associated mice; CoMA: Colonic microbiota-associated mice; SPF mice: Specific-pathogen-free mice; SI: Small intestine; LI: Large intestine. **Figure S5.** Heat map showing exogenous microbes that were successfully transplanted into jejunal microbiota-associated (JMA) mice. Jejunal and ileal samples of recipients were pooled into small-intestinal samples. Caecal, colonic, and fecal samples of recipients were pooled into large-intestinal samples. SI: Small intestine; LI: Large intestine. The values of color in the heat map represent the normalized relativea bundances of genera (Z-score normalization). *More abundant exogenous microbes colonized in the SI of JMA mice; ^#^More abundant exogenous microbes colonized in the LI of JMA mice. **Figure S6.** Heat map showing exogenous microbes that were successfully transplanted into ileal microbiota-associated (IMA) mice. Jejunal and ileal samples of recipients were pooled into small-intestinal samples. Caecal, colonic, and fecal samples of recipients were pooled into large-intestinal samples. SI: small intestine; LI: large intestine. The values of color in the heat map represent the normalized relativea bundances of genera (Z-score normalization). **Figure S7.** Heat map showing exogenous microbes that were successfully transplanted into cecal microbiota-associated (CeMA) mice. Jejunal and ileal samples of recipients were pooled into small-intestinal samples. Caecal, colonic, and fecal samples of recipients were pooled into large-intestinal samples. SI: small intestine; LI: large intestine. The values of color in the heat map represent the normalized relativea bundances of genera (Z-score normalization). *More abundant exogenous microbes colonized in the SI of CeMA mice; ^#^More abundant exogenous microbes colonized in the LI of CeMA mice. **Figure S8.** Heat map showing exogenous microbes that were successfully transplanted into colonic microbiota-associated (CoMA) mice. Jejunal and ileal samples of recipients were pooled into small-intestinal samples. Caecal, colonic, and fecal samples of recipients were pooled into large-intestinal samples. SI: small intestine; LI: large intestine. The values of color in the heat map represent the normalized relativea bundances of genera (Z-score normalization). *More abundant exogenous microbes colonized in the SI of CoMA mice; ^#^More abundant exogenous microbes colonized in the LI of CoMA mice. **Figure S9.** Differentially microbial functional profiles between SI and LI of recipient mice. Differentially microbial functional profiles between SI and LI of JMA mice (a), IMA mice (b), CeMA mice (c), and CoMA mice (d). Jejunal and ileal samples of recipients were pooled into small-intestinal samples. Caecal, colonic, and fecal samples of recipients were pooled into large-intestinal samples. Data are shown as means. JMA mice: Jejunal microbiota-associated mice; IMA: Ileal microbiota-associated mice; CeMA: Cecal microbiota-associated mice; CoMA: Colonic microbiota-associated mice; SI: Small intestine; LI: Large intestine. **Figure S10.** Gut microbiota structure of FMA mice, WIMA mice, and donor pigs. Principal coordinate analysis (PCoA) plots based on Bray-Curtis distances in fecal microbiota-associated mice, T5 (a), and whole-intestinal microbiota-associated mice, T6 (b). Principal coordinate analysis (PCoA) plots based on Jaccard distances in fecal microbiota-associated mice, T5 (c), and whole-intestinal microbiota-associated mice, T6 (d). D: Donor; J: Jejunum; I: Ileum; Ce: Cecum; Co: Colon; F: Feces; WI: Whole intestine. **Figure S11.** Total bacterial population in FMA and WIMA mice and their donors determined by quantitative PCR. Differences in the copy numbers of the total bacteria (log10 copies/g wet digesta) between the whole-intestine and feces of feces (a), between SI and LI of recipients (b) and between FMA and WIMA mice (c). Jejunal and ileal samples of mice were pooled into small-intestinal samples. Caecal, colonic, and fecal samples of mice were pooled into large-intestinal samples. FMA mice: Fecal microbiota-associated mice; WIMA mice: Whole-intestinal microbiota-associated mice; SI: Small intestine; LI: Large intestine. **Figure S12.** Heat map showing exogenous microbes that were successfully transplanted into fecal microbiota-associated (FMA) mice. Jejunal and ileal samples of recipients were pooled into small-intestinal samples. Caecal, colonic, and fecal samples of recipients were pooled into large-intestinal samples. SI: small intestine; LI: large intestine. The values of color in the heat map represent the normalized relativea bundances of genera (Z-score normalization). *More abundant exogenous microbes colonized in the SI of FMA mice; ^#^More abundant exogenous microbes colonized in the LI of FMA mice. **Figure S13.** Heat map showing exogenous microbes that were successfully transplanted into whole-intestinal microbiota-associated (WIMA) mice. Jejunal and ileal samples of recipients were pooled into small-intestinal samples. Caecal, colonic, and fecal samples of recipients were pooled into large-intestinal samples. SI: small intestine; LI: large intestine. The values of color in the heat map represent the normalized relativea bundances of genera (Z-score normalization). *More abundant exogenous microbes colonized in the SI of WIMA mice; ^#^More abundant exogenous microbes colonized in the LI of WIMA mice. **Figure S14.** Differentially microbial functional profiles of FMA and WIMA mice. Differential abundant KEGG pathways in small-intestinal samples (a) and large-intestinal samples (b) are plotted. Jejunal and ileal samples of recipients were pooled into small-intestinal samples. Caecal, colonic, and fecal samples of recipients were pooled into large-intestinal samples. Data are shown as means. FMA mice: Fecal microbiota-associated mice; WIMA mice: Whole-intestinal microbiota-associated mice; SI: Small intestine; LI: Large intestine. **Figure S15.** The development of small-intestinal epithelial morphology of GF, recipient, and SPF mice. Differences in the villus height, crypt depth, the number of apoptotic positive cells, and the number of acidic and neutral mucins in the jejunum (a-e) and ileum (f-j) among groups are presented. GF: Germ-free mice; JMA mice: Jejunal microbiota-associated mice; IMA mice: Ileal microbiota-associated mice; CeMA mice: Cecal microbiota-associated mice; CoMA mice: Colonic microbiota-associated mice; SPF mice: Specific-pathogen-free mice; J: Jejunum; I: Ileum. Data are shown as mean±SEM. **Figure S16.** Plasma inflammatory profiles of GF, recipient, and SPF mice. Differences in concentrations of IFN-γ (a), IL-1β (b), IL-5 (c), IL-6 (d), IL-12p70 (e), KC/GRO (f), TNF-α (g), IL-2 (h), IL-4 (i), and IL-10 (j) among different groups of mice are presented. Data are shown as mean±SEM. GF: Germ-free mice; JMA mice: Jejunal microbiota-associated mice; IMA mice: Ileal microbiota-associated mice; CeMA mice: Cecal microbiota-associated mice; CoMA mice: Colonic microbiota-associated mice; SPF mice: Specific-pathogen-free mice. Data are shown as mean±SEM.*P < 0.05, **P < 0.01, ***P < 0.001.

**Additional file 2: Supplementary tables. Table S1a.** Donor microbes that were successfully transplanted into jejunal microbiota-associated (JMA) mice. SI: small intestine; LI: large intestine. * More abundant exogenous microbes colonized in the SI of JMA mice; # More abundant exogenous microbes colonized in the LI of JMA mice. **Table S1b.** Donor microbes that were successfully transplanted into ileal microbiota-associated (IMA) mice. SI: small intestine; LI: large intestine. **Table S1c. **Donor microbes that were successfully transplanted into cecal microbiota-associated (CeMA) mice. SI: small intestine; LI: large intestine. * More abundant exogenous microbes colonized in the SI of CeMA mice; # More abundant exogenous microbes colonized in the LI of CeMA mice. **Table S1d.** Donor microbes that were successfully transplanted into colonic microbiota-associated (CoMA) mice. SI: small intestine; LI: large intestine. * More abundant exogenous microbes colonized in the SI of CoMA mice; # More abundant exogenous microbes colonized in the LI of CoMA mice. Table S1d. Donor microbes that were successfully transplanted into colonic microbiota-associated (CoMA) mice. SI: small intestine; LI: large intestine. * More abundant exogenous microbes colonized in the SI of CoMA mice; # More abundant exogenous microbes colonized in the LI of CoMA mice. **Table S1e.** Donor microbes that were successfully transplanted into fecal microbiota-associated (FMA) mice. SI: small intestine; LI: large intestine. * More abundant exogenous microbes colonized in the SI of FMA mice; # More abundant exogenous microbes colonized in the LI of FMA mice. **Table S1f.** Donor microbes that were successfully transplanted into whole-intestinal microbiota-associated (WIMA) mice.SI: small intestine; LI: large intestine. * More abundant exogenous microbes colonized in the SI of WIMA mice; # More abundant exogenous microbes colonized in the LI of WIMA mice.

## Data Availability

The datasets supporting the conclusions of this article are available in the NCBI Sequence Read Archive (SRA) repository under accession number PRJNA593023 (available on 02 January 2021).

## Abbreviations

GI-tract: Gatrointestinal tract
FMT: Fecal microbiota transplantation
SI: Small intestine
LI: Large intestine
WIMT: Whole-intestinal microbiota transplantation
SPF: Specific-pathogen-free
JMA: Jejunal microbiota-associated
IMA: Ileal microbiota-associated
CeMA: Cecal microbiota-associated
CoMA: Colonic microbiota-associated
FMA: Fecal microbiota-associated
WIMA: Whole-intestinal microbiota-associated
ASVs: Amplicon sequence variants
PERMANOVA: Permutational multivariate analysis of variance
LDA: Linear discriminant analysis
LEfSe: Linear discriminant analysis effect size
FDR: False discovery rate
SEM: Standard error of the mean
PCoA: Principal coordinates analysis
SCFAs: Short-chain fatty acids
