# Supplementary material for: Spatial heterogeneity of bacterial colonization across different gut segments following inter-species microbiota transplantation
Source: Microbiome. 2020 Nov 18;8:161. doi: 10.1186/s40168-020-00917-7 (PMC7677849; doi:10.1186/s40168-020-00917-7)

**Supplementary figures**

**Figure S1. Gut microbiota structure of recipient mice, SPF mice, and donor pigs.** Principal coordinate analysis (PCoA) plots based on Bray-Curtis distances in jejunal microbiota-associated mice, T1 **(a)**, ileal microbiota-associated mice, T2 **(b)**, cecal microbiota-associated mice, T3 **(c)**, colonic microbiota-associated mice, T4 **(d)**, and specific-pathogen-free mice **(e)**. D: Donor; J: Jejunum; I: Ileum; Ce: Cecum; Co: Colon; F: Feces.


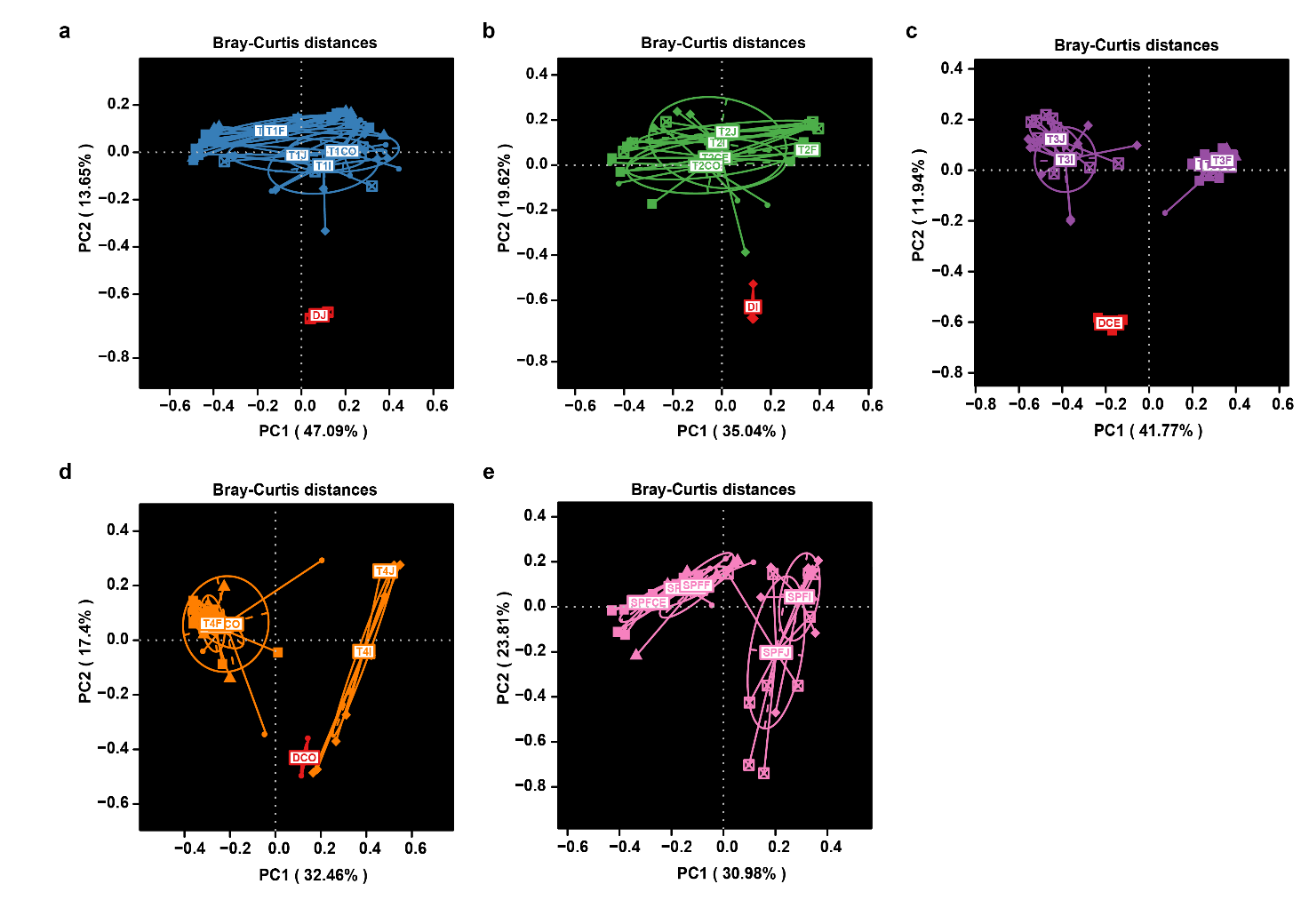


**Figure S2. Gut microbiota structure of recipient mice, SPF mice, and donor pigs.** Principal coordinate analysis (PCoA) plots based on Jaccard distances in jejunal microbiota-associated mice, T1 **(a)**, ileal microbiota-associated mice, T2 **(b)**, cecal microbiota-associated mice, T3 **(c)**, colonic microbiota-associated mice, T4 **(d)**, and specific-pathogen-free mice mice **(e)**. D: Donor; J: Jejunum; I: Ileum; Ce: Cecum; Co: Colon; F: Feces.

**
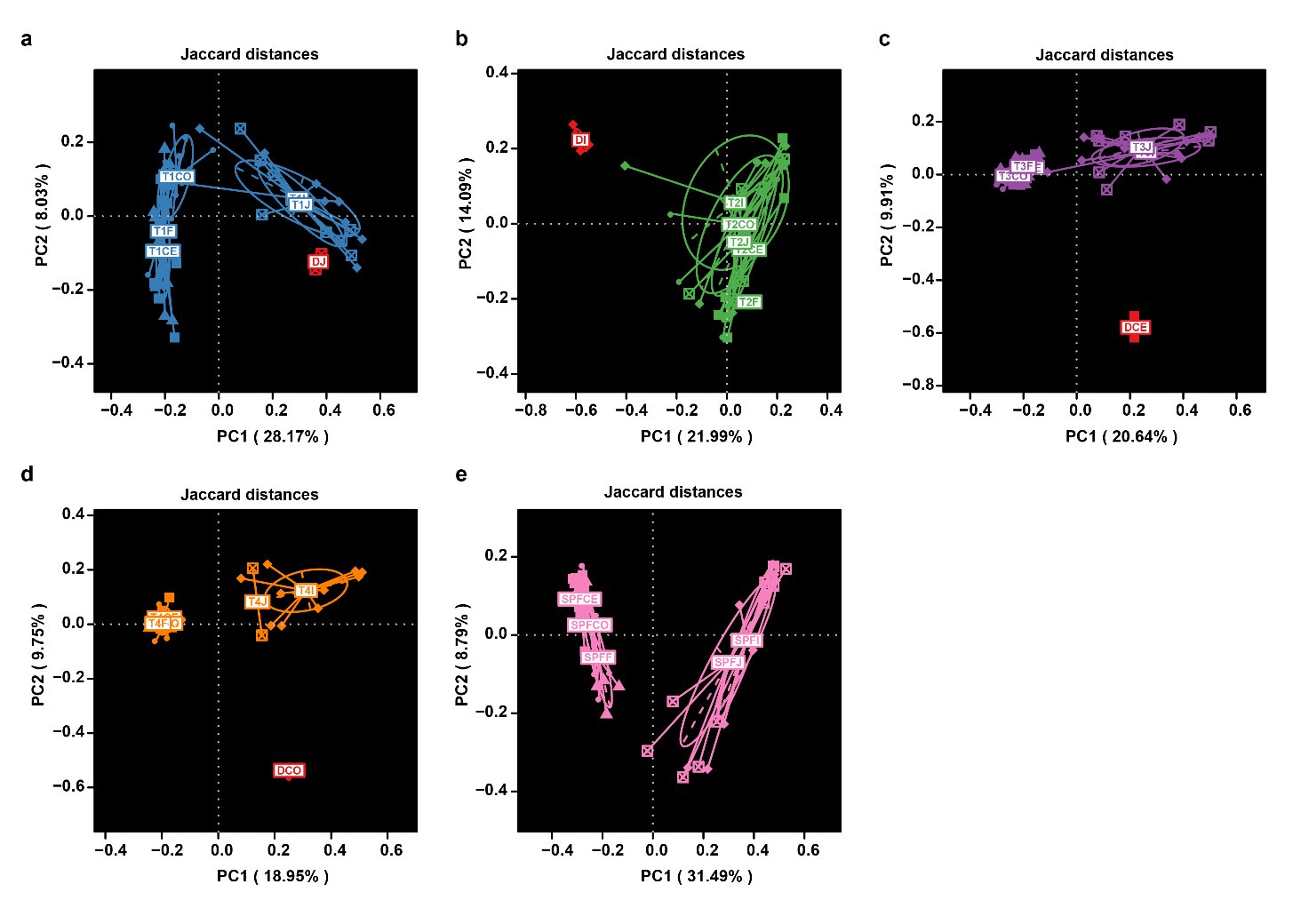
**

**Figure S3. Gut microbiota composition among different groups of donors and mice**. Abundant phyla (a), families (b), and genera (c) in the gut microbiota of different groups of donors and mice. Only genera with average relative abundance greater than 1% were shown. Data are shown as means in each group, D: Donor; J: Jejunum; I: Ileum; Ce: Cecum; Co: Colon; F: Feces; WI: Whole intestine; T1: Jejunal microbiota-associated mice; T2: Ileal microbiota-associated mice; T3: Cecal microbiota-associated mice; T4: Colonic microbiota-associated mice; T5: Fecal microbiota-associated mice; T6: Whole-intestinal microbiota-associated mice; SPF: Specific-pathogen-free mice.


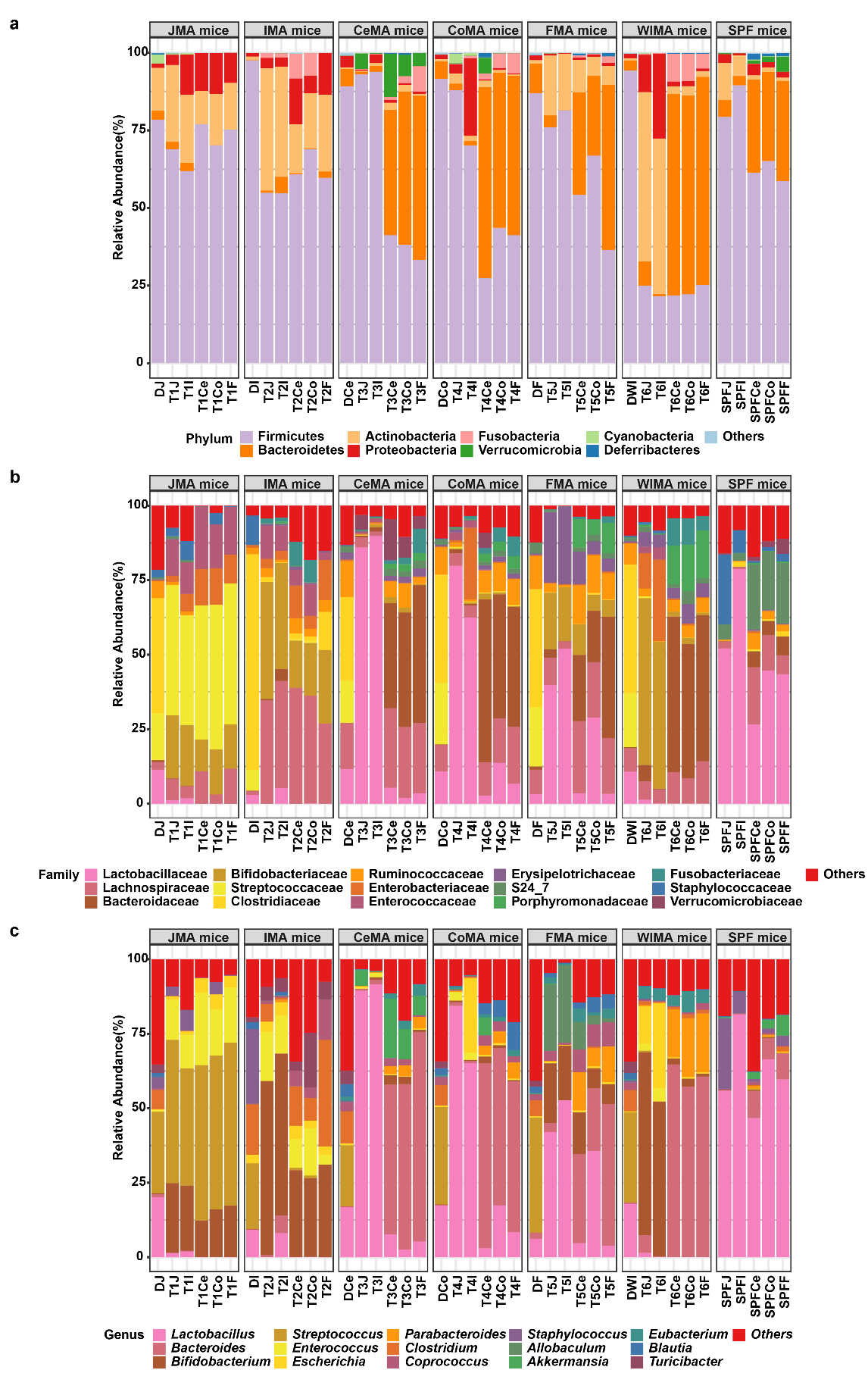


**Figure S4. Total bacterial population in recipient mice, and SPF mice, that were determined by quantitative PCR.** Differences in the copy numbers of the total bacteria (log10 copies/g wet digesta) among donors (**a**), among different groups (**b**) and between SI and LI of recipients (**c**). Jejunal and ileal samples of recipients were pooled into small-intestinal samples. Caecal, colonic, and fecal samples of recipients were pooled into large-intestinal samples. JMA mice: Jejunal microbiota-associated mice; IMA: Ileal microbiota-associated mice; CeMA: Cecal microbiota-associated mice; CoMA: Colonic microbiota-associated mice; SPF mice: Specific-pathogen-free mice; SI: Small intestine; LI: Large intestine

**
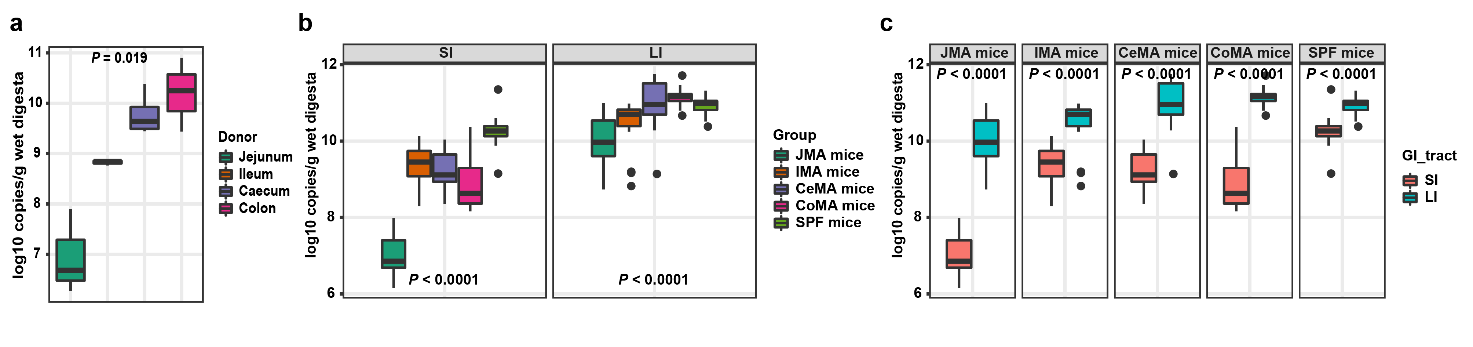
**

**Figure S5. Heat map showing exogenous microbes that were successfully transplanted into jejunal microbiota-associated (JMA) mice.** Jejunal and ileal samples of recipients were pooled into small-intestinal samples. Caecal, colonic, and fecal samples of recipients were pooled into large-intestinal samples. SI: Small intestine; LI: Large intestine. The values of color in the heat map represent the normalized relativea bundances of genera (Z-score normalization). *More abundant exogenous microbes colonized in the SI of JMA mice; ^#^More abundant exogenous microbes colonized in the LI of JMA mice.


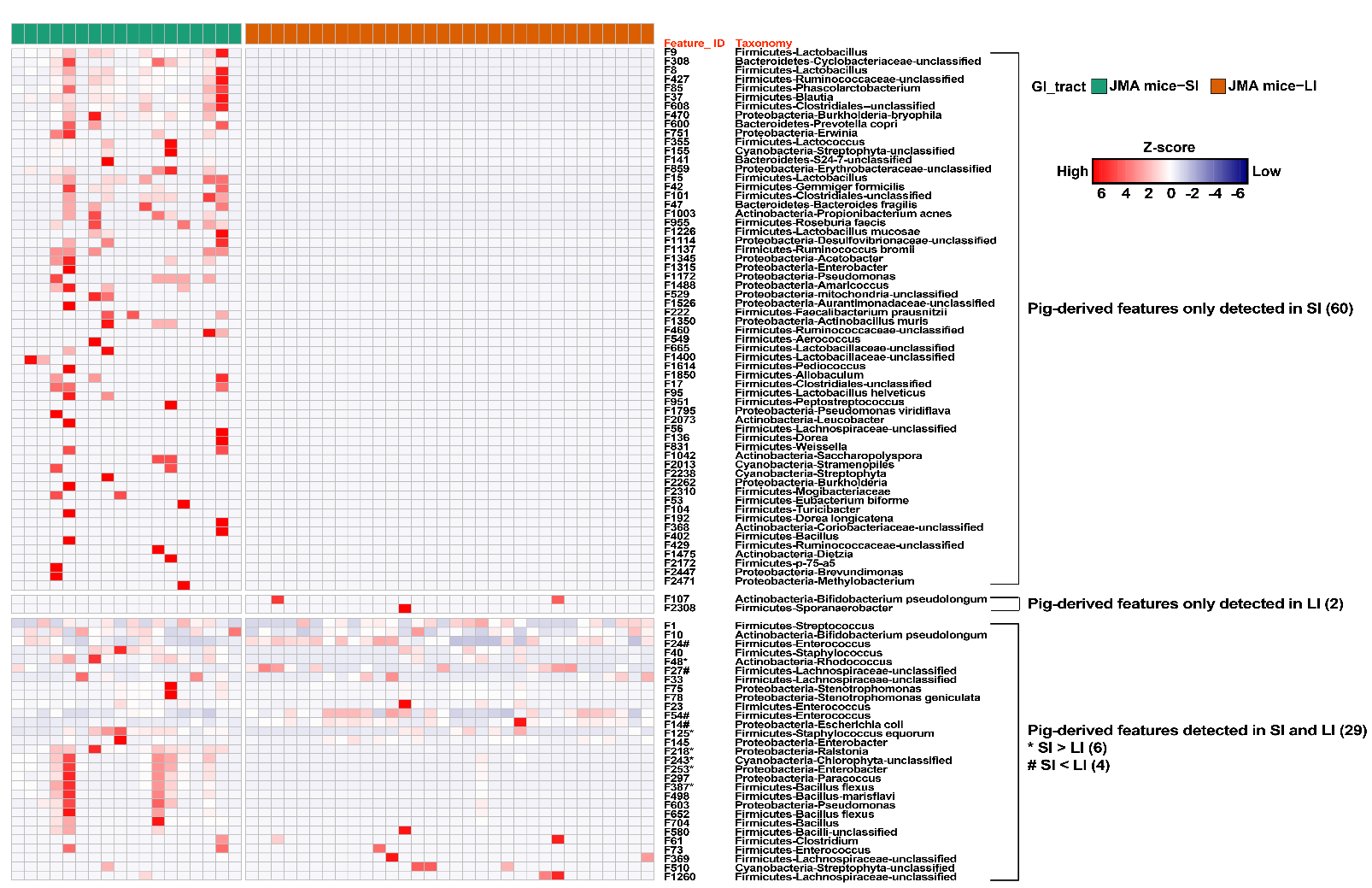


**Figure S6. Heat map showing exogenous microbes that were successfully transplanted into ileal microbiota-associated (IMA) mice.** Jejunal and ileal samples of recipients were pooled into small-intestinal samples. Caecal, colonic, and fecal samples of recipients were pooled into large-intestinal samples. SI: small intestine; LI: large intestine. The values of color in the heat map represent the normalized relativea bundances of genera (Z-score normalization).


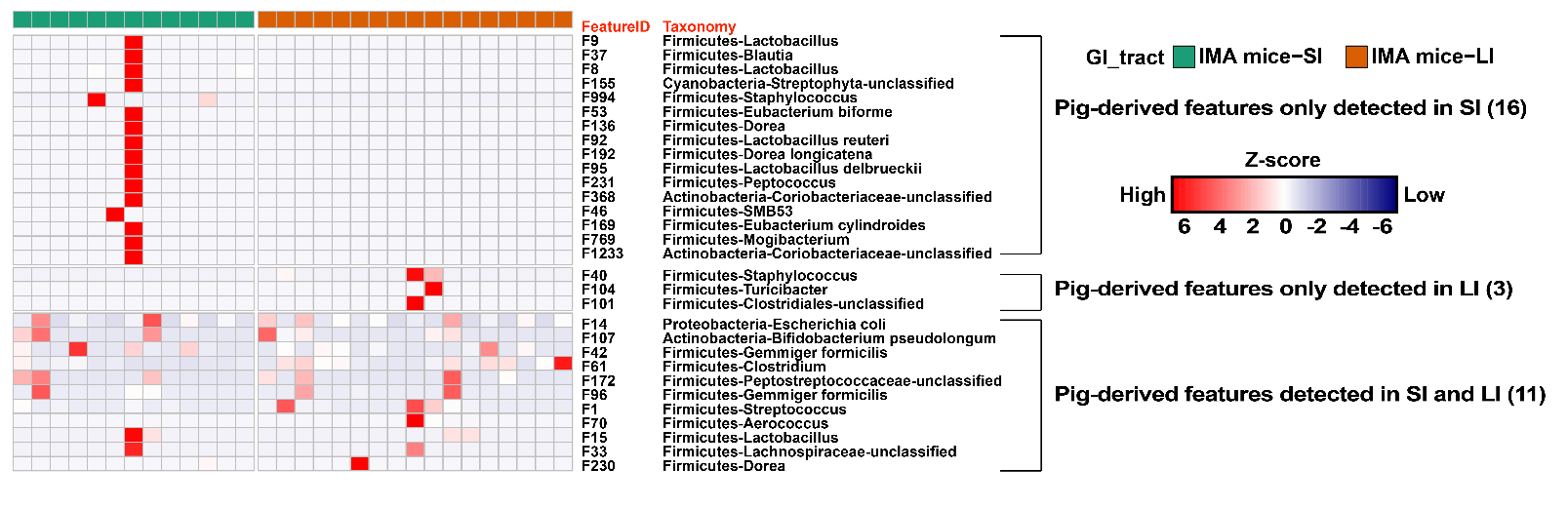


**Figure S7. Heat map showing exogenous microbes that were successfully transplanted into cecal microbiota-associated (CeMA) mice.** Jejunal and ileal samples of recipients were pooled into small-intestinal samples. Caecal, colonic, and fecal samples of recipients were pooled into large-intestinal samples. SI: small intestine; LI: large intestine. The values of color in the heat map represent the normalized relativea bundances of genera (Z-score normalization). *More abundant exogenous microbes colonized in the SI of CeMA mice; ^#^More abundant exogenous microbes colonized in the LI of CeMA mice.


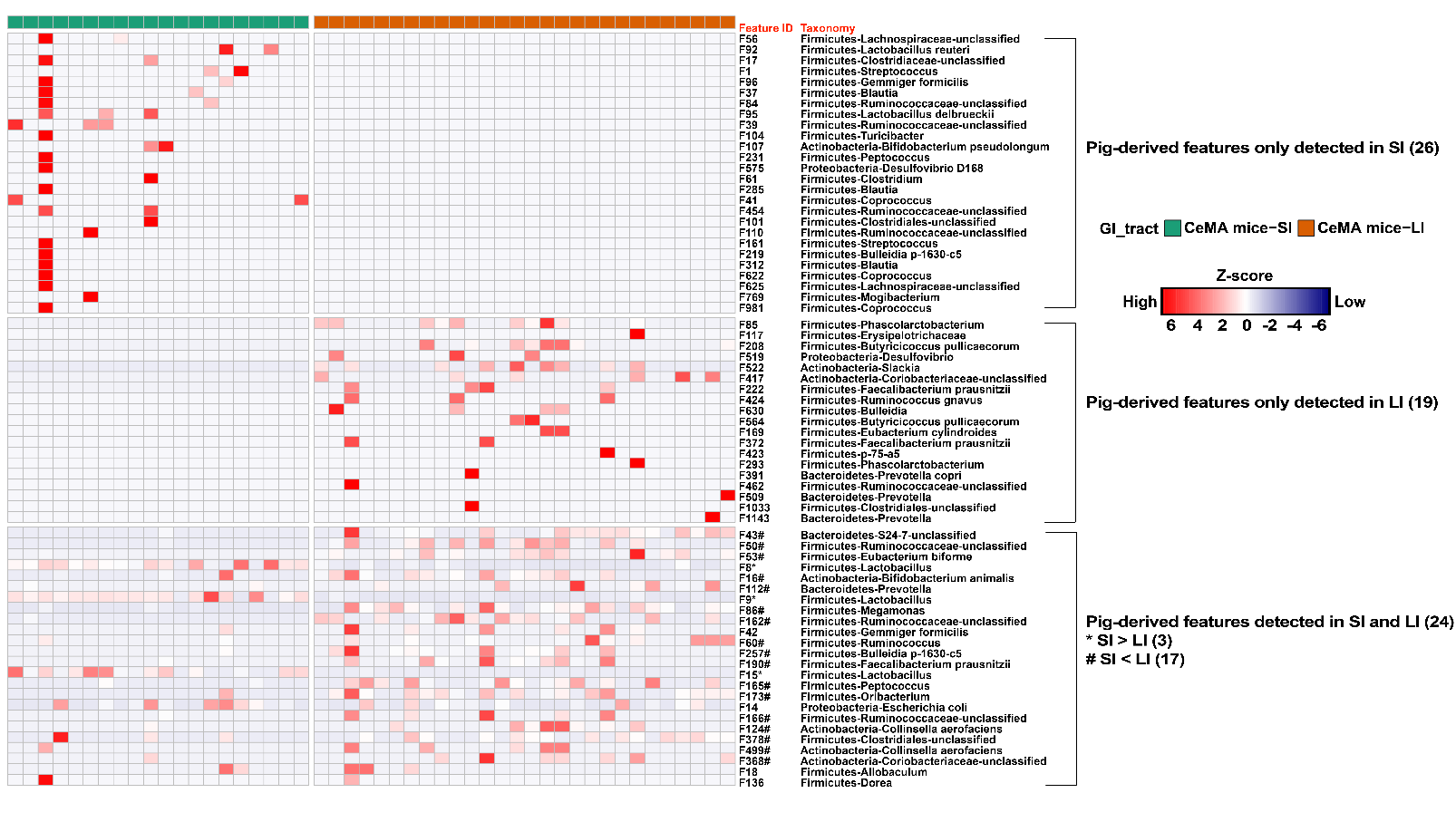


**Figure S8. Heat map showing exogenous microbes that were successfully transplanted into colonic microbiota-associated (CoMA) mice.** Jejunal and ileal samples of recipients were pooled into small-intestinal samples. Caecal, colonic, and fecal samples of recipients were pooled into large-intestinal samples. SI: small intestine; LI: large intestine. The values of color in the heat map represent the normalized relativea bundances of genera (Z-score normalization). *More abundant exogenous microbes colonized in the SI of CoMA mice; ^#^More abundant exogenous microbes colonized in the LI of CoMA mice.


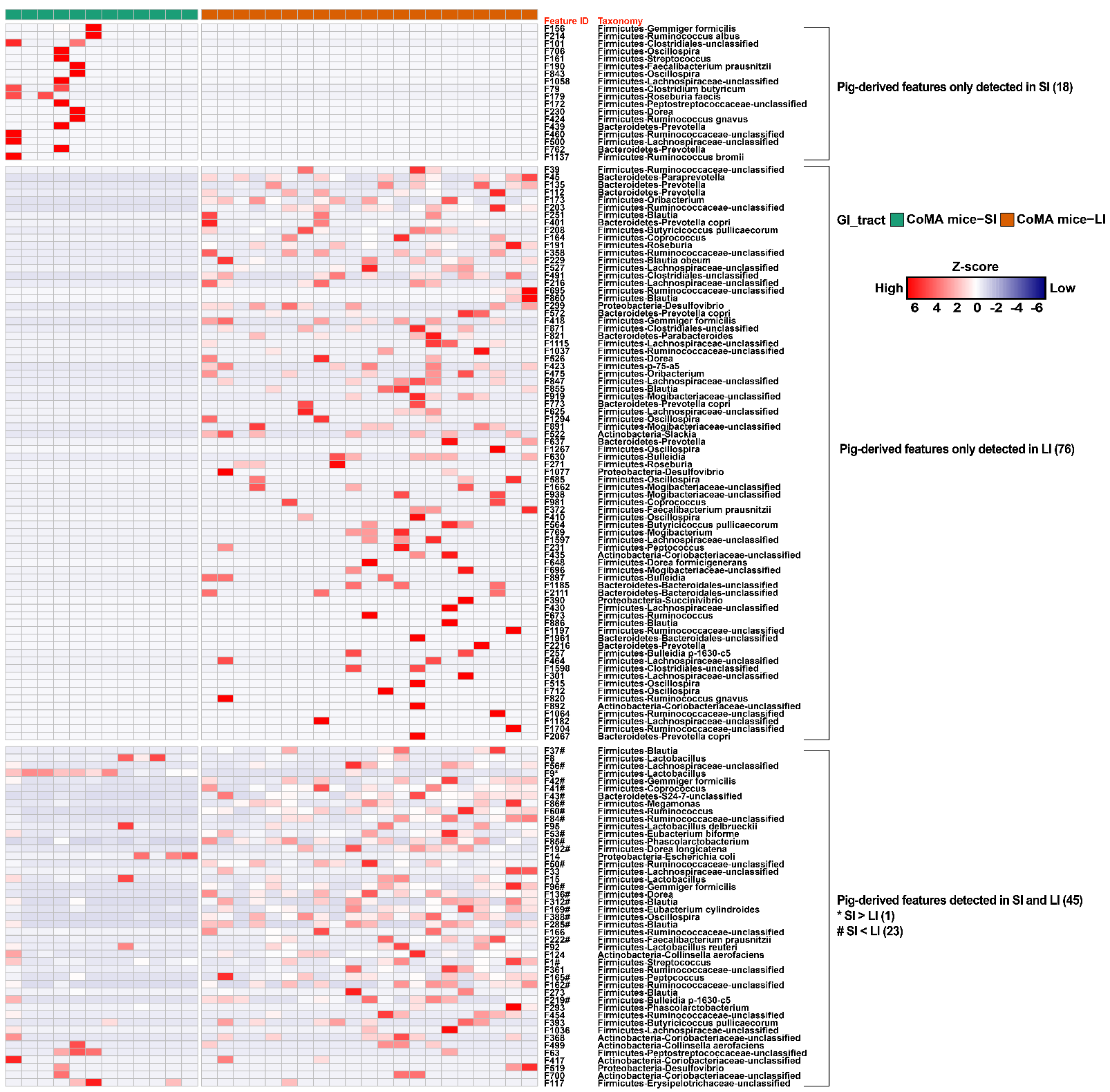


**Figure S9. Differentially microbial functional profiles between SI and LI of** **recipient mice.** Differentially microbial functional profiles between SI and LI of JMA mice (**a**), IMA mice (**b**), CeMA mice (**c**), and CoMA mice (**d**). Jejunal and ileal samples of recipients were pooled into small-intestinal samples. Caecal, colonic, and fecal samples of recipients were pooled into large-intestinal samples. Data are shown as means. JMA mice: Jejunal microbiota-associated mice; IMA: Ileal microbiota-associated mice; CeMA: Cecal microbiota-associated mice; CoMA: Colonic microbiota-associated mice; SI: Small intestine; LI: Large intestine.

**
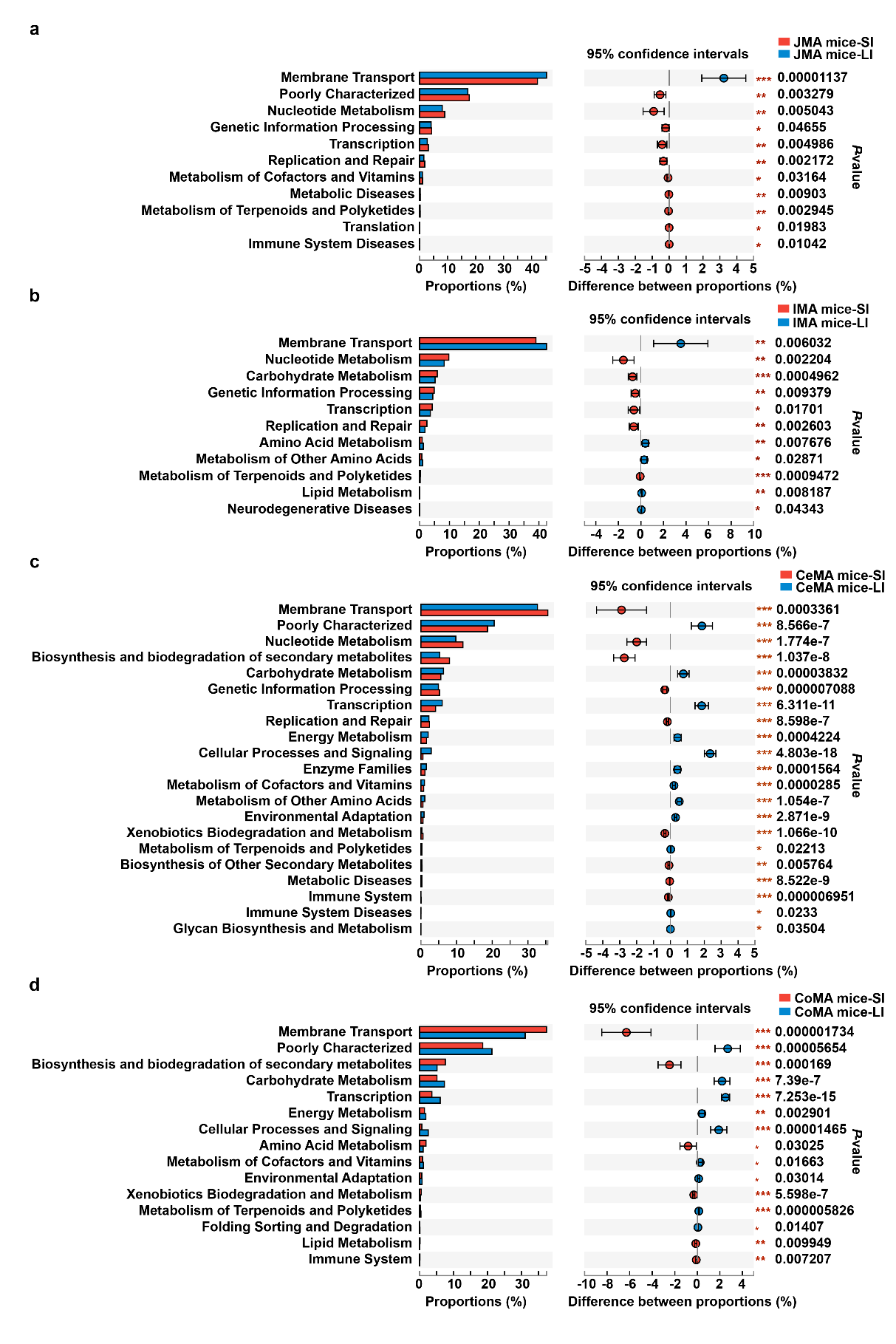
**

**Figure S10. Gut microbiota structure of FMA mice, WIMA mice, and donor pigs.** Principal coordinate analysis (PCoA) plots based on Bray-Curtis distances in fecal microbiota-associated mice, T5 **(a)**, and whole-intestinal microbiota-associated mice, T6 **(b)**. Principal coordinate analysis (PCoA) plots based on Jaccard distances in fecal microbiota-associated mice, T5 **(c)**, and whole-intestinal microbiota-associated mice, T6 **(d).** D: Donor; J: Jejunum; I: Ileum; Ce: Cecum; Co: Colon; F: Feces; WI: Whole intestine.

**
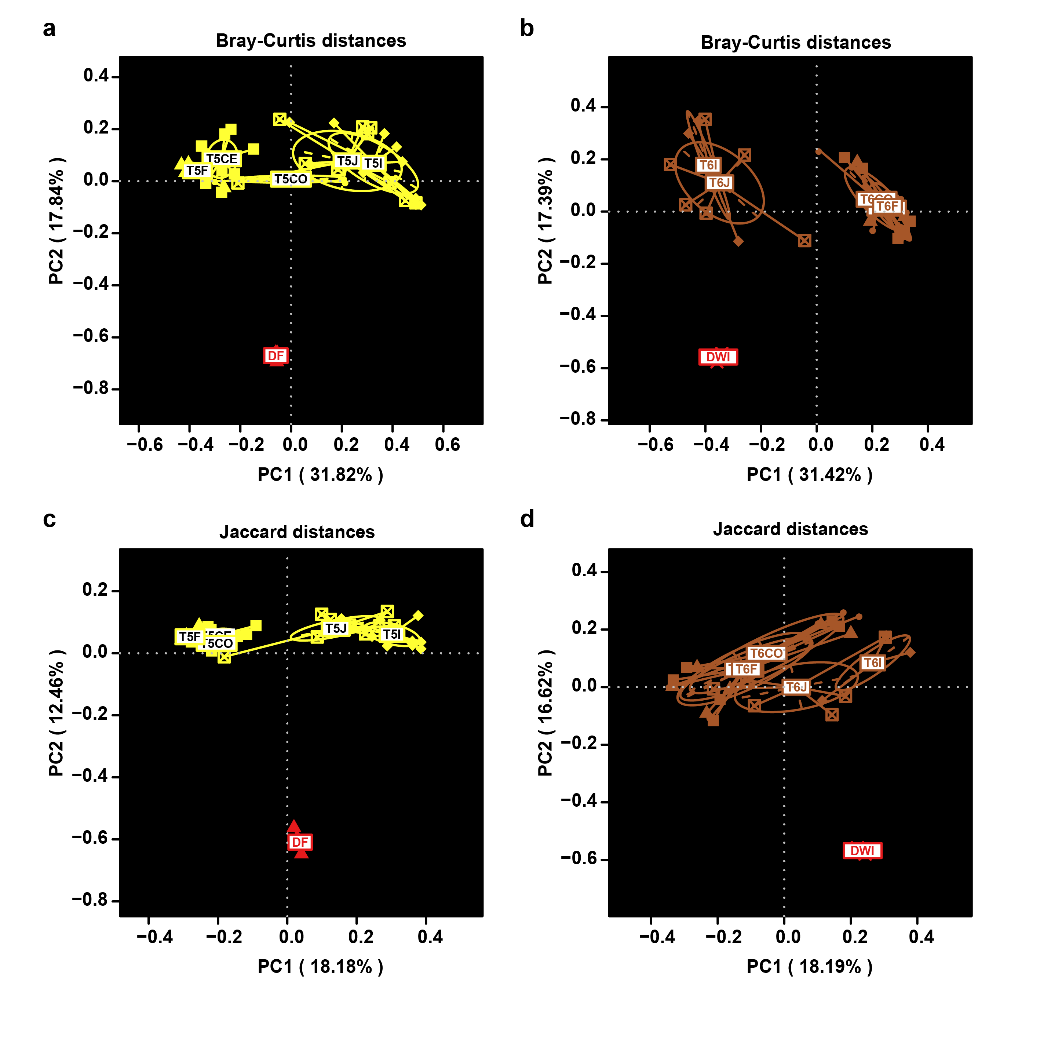
**

**Figure S11.** **Total bacterial population in FMA and WIMA mice and their donors determined by quantitative PCR.** Differences in the copy numbers of the total bacteria (log10 copies/g wet digesta) between the whole-intestine and feces of feces (**a**), between SI and LI of recipients (**b**) and between FMA and WIMA mice (**c**). Jejunal and ileal samples of mice were pooled into small-intestinal samples. Caecal, colonic, and fecal samples of mice were pooled into large-intestinal samples. FMA mice: Fecal microbiota-associated mice; WIMA mice: Whole-intestinal microbiota-associated mice; SI: Small intestine; LI: Large intestine

**
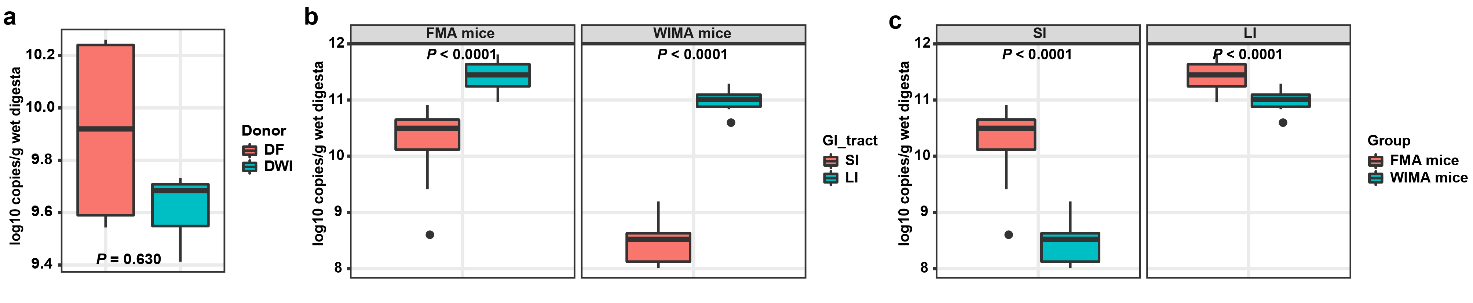
**

**Figure S12. Heat map showing exogenous microbes that were successfully transplanted into fecal microbiota-associated (FMA) mice.** Jejunal and ileal samples of recipients were pooled into small-intestinal samples. Caecal, colonic, and fecal samples of recipients were pooled into large-intestinal samples. SI: small intestine; LI: large intestine. The values of color in the heat map represent the normalized relativea bundances of genera (Z-score normalization). *More abundant exogenous microbes colonized in the SI of FMA mice; ^#^More abundant exogenous microbes colonized in the LI of FMA mice.


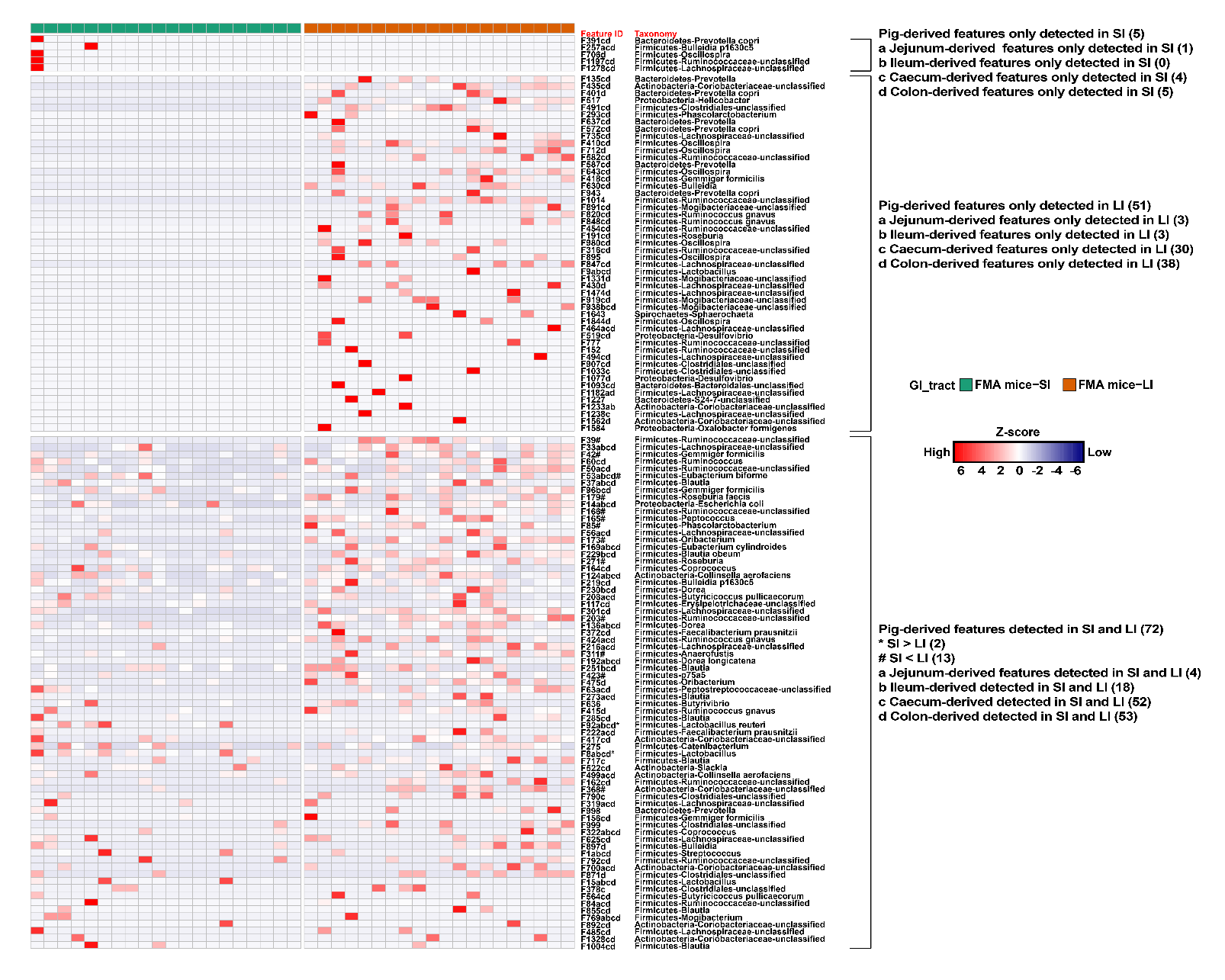


**Figure S13. Heat map showing exogenous microbes that were successfully transplanted into whole-intestinal microbiota-associated (WIMA) mice.** Jejunal and ileal samples of recipients were pooled into small-intestinal samples. Caecal, colonic, and fecal samples of recipients were pooled into large-intestinal samples. SI: small intestine; LI: large intestine. The values of color in the heat map represent the normalized relativea bundances of genera (Z-score normalization). *More abundant exogenous microbes colonized in the SI of WIMA mice; ^#^More abundant exogenous microbes colonized in the LI of WIMA mice.


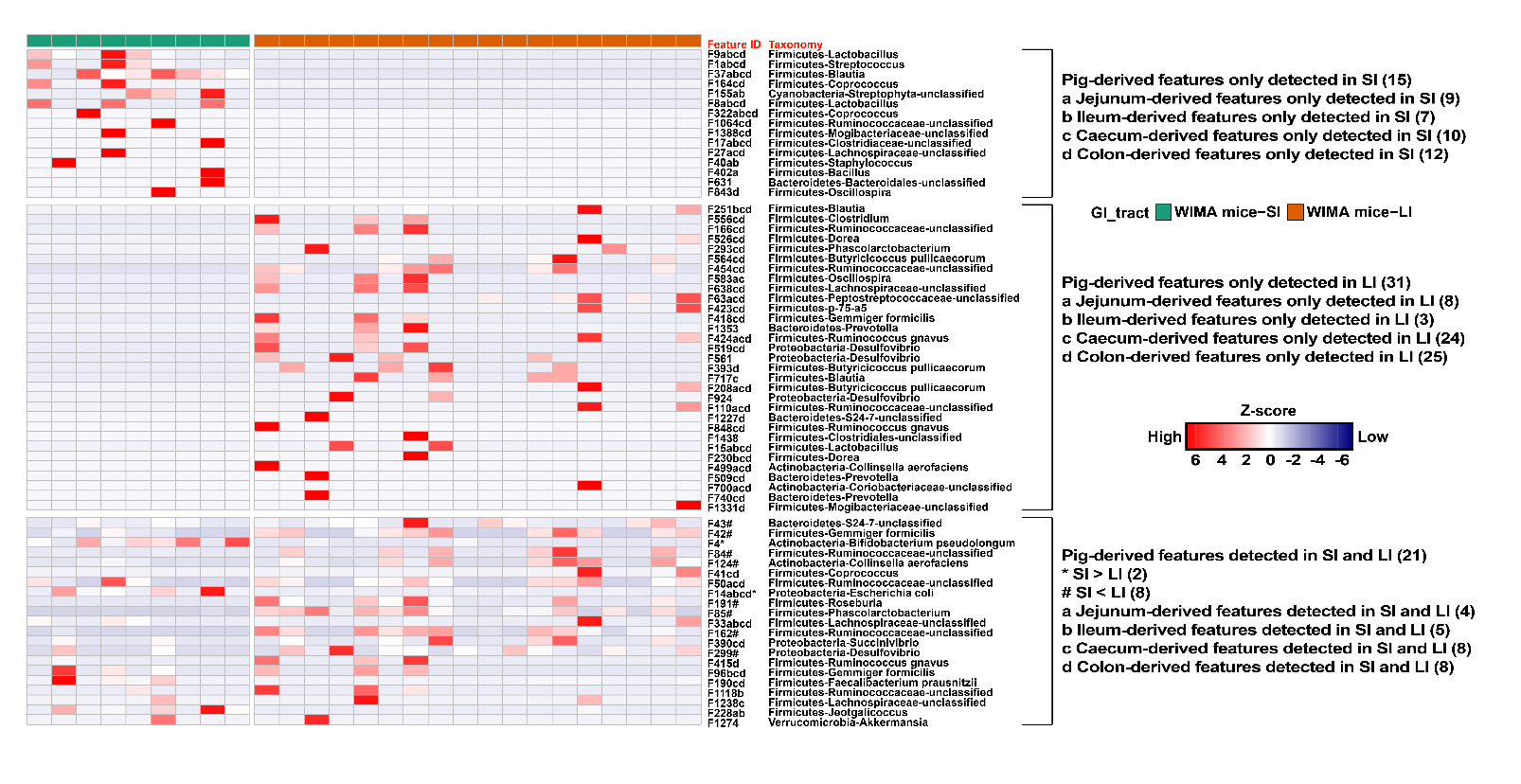


**Figure S14. Differentially microbial functional profiles of FMA and WIMA mice.** Differential abundant KEGG pathways in small-intestinal samples (**a**) and large-intestinal samples (**b**) are plotted. Jejunal and ileal samples of recipients were pooled into small-intestinal samples. Caecal, colonic, and fecal samples of recipients were pooled into large-intestinal samples. Data are shown as means. FMA mice: Fecal microbiota-associated mice; WIMA mice: Whole-intestinal microbiota-associated mice; SI: Small intestine; LI: Large intestine.


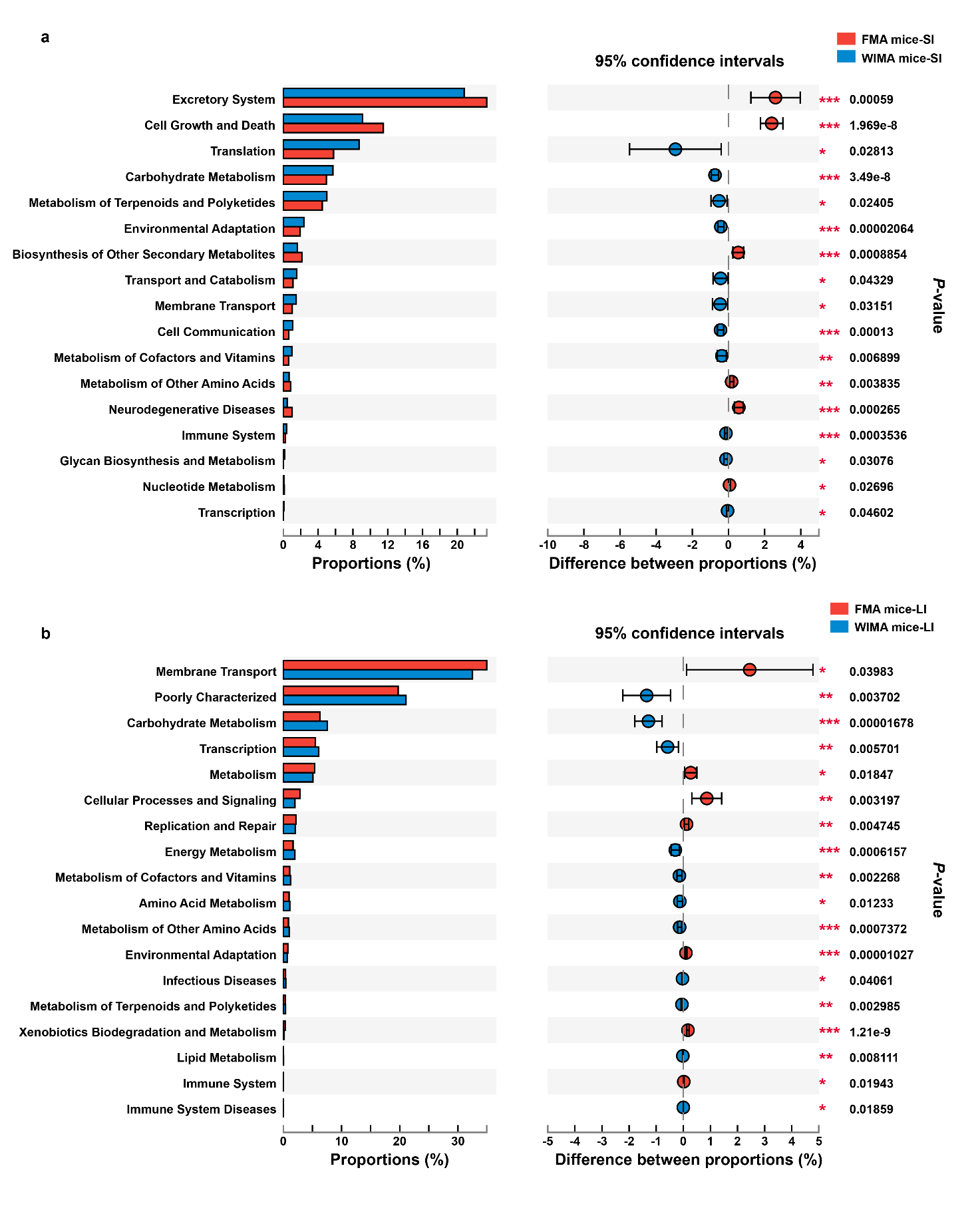


**Figure S15. The development of small-intestinal epithelial morphology of GF, recipient, and SPF mice.** Differences in the villus height, crypt depth, the number of apoptotic positive cells, and the number of acidic and neutral mucins in the jejunum (**a-e**) and ileum (**f-j**) among groups are presented. GF: Germ-free mice; JMA mice: Jejunal microbiota-associated mice; IMA mice: Ileal microbiota-associated mice; CeMA mice: Cecal microbiota-associated mice; CoMA mice: Colonic microbiota-associated mice; SPF mice: Specific-pathogen-free mice; J: Jejunum; I: Ileum. Data are shown as mean±SEM.


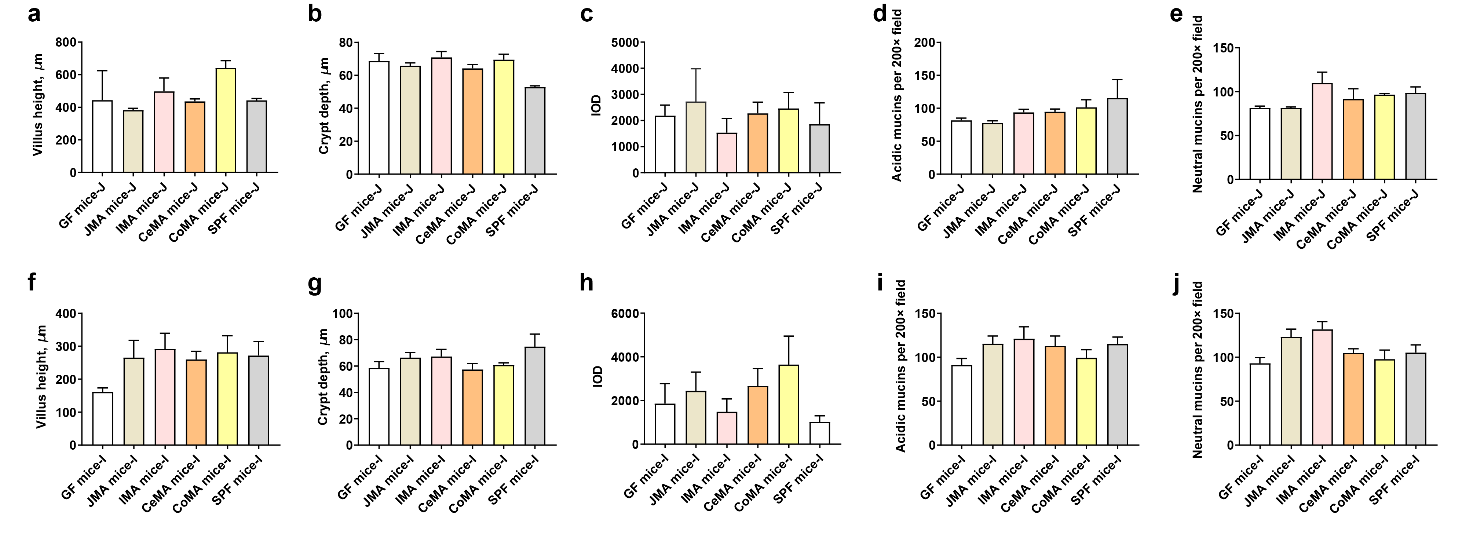


**Figure S16. Plasma inflammatory profiles of GF, recipient, and SPF mice.** Differences in concentrations of IFN-γ (**a**), IL-1β (**b**), IL-5 (**c**), IL-6 (**d**), IL-12p70 (**e**), KC/GRO (**f**), TNF-α (**g**), IL-2 (**h**), IL-4 (**i**), and IL-10 (**j**) among different groups of mice are presented. Data are shown as mean±SEM. GF: Germ-free mice; JMA mice: Jejunal microbiota-associated mice; IMA mice: Ileal microbiota-associated mice; CeMA mice: Cecal microbiota-associated mice; CoMA mice: Colonic microbiota-associated mice; SPF mice: Specific-pathogen-free mice. Data are shown as mean±SEM.**P* < 0.05, ***P* < 0.01, ****P* < 0.001.


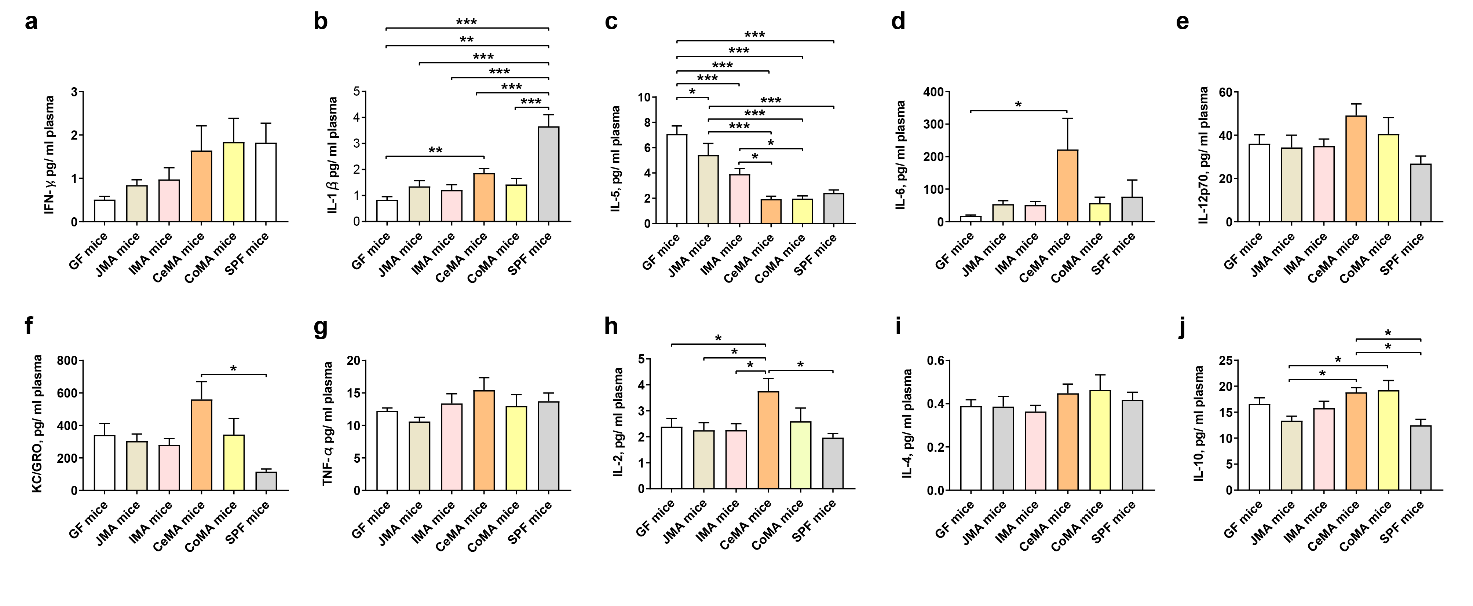

Supplement: Supplementary file 2 — Additional file 1: Supplementary figures. Figure S1. Gut microbiota structure of recipient mice, SPF mice, and donor pigs. Principal coordinate analysis (PCoA) plots based on Bray-Curtis distances in jejunal microbiota-associated mice, T1 (a), ileal microbiota-associated mice, T2 (b), cecal microbiota-associated mice, T3 (c), colonic microbiota-associated mice, T4 (d), and specific-pathogen-free mice (e). D: Donor; J: Jejunum; I: Ileum; Ce: Cecum; Co: Colon; F: Feces. Figure S2. Gut microbiota structure of recipient mice, SPF mice, and donor pigs. Principal coordinate analysis (PCoA) plots based on Jaccard distances in jejunal microbiota-associated mice, T1 (a), ileal microbiota-associated mice, T2 (b), cecal microbiota-associated mice, T3 (c), colonic microbiota-associated mice, T4 (d), and specific-pathogen-free mice (e). D: Donor; J: Jejunum; I: Ileum; Ce: Cecum; Co: Colon; F: Feces. Figure S3. Gut microbiota composition among different groups of donors and mice. Abundant phyla (a), families (b), and genera (c) in the gut microbiota of different groups of donors and mice. Only genera with average relative abundance greater than 1% were shown. Data are shown as means in each group, D: Donor; J: Jejunum; I: Ileum; Ce: Cecum; Co: Colon; F: Feces; WI: Whole intestine; T1: Jejunal microbiota-associated mice; T2: Ileal microbiota-associated mice; T3: Cecal microbiota-associated mice; T4: Colonic microbiota-associated mice; T5: Fecal microbiota-associated mice; T6: Whole-intestinal microbiota-associated mice; SPF: Specific-pathogen-free mice. Figure S4. Total bacterial population in recipient mice, and SPF mice, that were determined by quantitative PCR. Differences in the copy numbers of the total bacteria (log10 copies/g wet digesta) among donors (a), among different groups (b) and between SI and LI of recipients (c). Jejunal and ileal samples of recipients were pooled into small-intestinal samples. Cecal, colonic, and fecal samples of recipients were pooled into large [file 40168_2020_917_MOESM1_ESM.docx]
